# Free-form catenary-inspired meta-couplers for ultra-high or broadband vertical coupling

**DOI:** 10.1515/nanoph-2024-0566

**Published:** 2025-01-08

**Authors:** Tianqu Chen, Mingfeng Xu, Mingbo Pu, Xi Tang, Yuhan Zheng, Qingji Zeng, Yuting Xiao, Yingli Ha, Yinghui Guo, Fei Zhang, Nan Chi, Xiangang Luo

**Affiliations:** Department of Communication, Science and Engineering, Fudan University, Shanghai 200438, China; National Key Laboratory of Optical Field Manipulation Science and Technology, Chinese Academy of Sciences, Chengdu 610209, China; State Key Laboratory of Optical Technologies on Nano-Fabrication and Micro-Engineering, Institute of Optics and Electronics, Chinese Academy of Sciences, Chengdu 610209, China; Research Center on Vector Optical Fields, Institute of Optics and Electronics, Chinese Academy of Sciences, Chengdu 610209, China; College of Materials Sciences and Opto-Electronic Technology, University of Chinese Academy of Sciences, Beijing 100049, China

**Keywords:** integrated photonics, topology optimization, catenary, nanophotonics

## Abstract

Metasurface-assisted waveguide couplers, or meta-couplers, innovatively link free-space optics with on-chip devices, offering flexibility for polarization and wavelength (de)multiplexing, mode-selective coupling, and guided mode manipulation. However, conventional meta-couplers still face challenges with low coupling efficiency and narrow bandwidth due to critical near-field coupling caused by waveguide constraints and unit-cell–based design approach, which cannot be accurately addressed using traditional design methods. In this paper, quasi-continuous dielectric catenary arrays are first employed to enhance efficiency and bandwidth by addressing adjacent coupling issues of discrete metasurface. Then, diffraction analysis demonstrates that the performance of forward-designed couplers is hindered by spurious diffraction orders and destructive interference. To further enhance performance, an adjoint-based topology optimization algorithm is utilized to customize electric near-field, which can effectively suppress spurious diffraction orders and destructive near-field interference, achieving ultra-high coupling efficiency of 93 % with 16.7 dB extinction ratios at 1,550 nm. Additionally, a broadband meta-coupler exceeds 350 nm bandwidth with 50 % average coupling efficiency across O- to L-bands using multiobjective optimization. These high-performance devices may render them suitable for applications in optical communications, sensing, and nonlinear optics. Moreover, the inverse design method shows potential for improving the performance of various metasurface-integrated on-chip devices.

## 1 Introduction

Silicon photonics integrated on-chip devices are a powerful platform for realizing optical processors and communications due to their low cost and high compactness [1]. Among them, the coupling devices play a crucial role in bridging free-space light and on-chip devices. Huge progress has been achieved in vertical coupling techniques during the last decade [2]. However, overcoming the limitations in coupling efficiency (CE) and bandwidth (BW) of surface grating couplers remains challenging due to their inherent diffraction characteristics [3]. Various strategies have been proposed to enhance the performance of couplers, including improving coupling efficiency (CE), bandwidth (BW), extinction ratio (ER), and enabling multifunctional integration. To reduce the coupling loss, the buffer layer is often utilized to create constructive interference conditions for the reflected waves [4]. A plasmonic grating structure, in conjunction with a metal buffer deflection layer, is proposed as an effective and broadband solution for plasmonic slot waveguide couplers [5]. This design achieves a maximum CE of 72 % and a 3 dB BW of 350 nm. Inverse design method, such as the field overlap optimization algorithm, is utilized to maximize the peak CE of dual layer coupler, resulting in an impressive achievement of 88 % [6]. Additionally, total internal reflection (TIR) 3D structures [7], [8] or curved mirror-based polymer [9] couplers are utilized for the ultra-broadband and high efficiency coupling implementation. The best CE and BW records of 3D couplers are 80 % for 300 nm BW [10] and 16 % for 970 nm BW [8].

However, previous grating or polymer structure–based couplers suffer from low polarization selectivity and a lack of mode modulation capabilities. As technology progresses, advances in imaging sensors, augmented reality (AR), and virtual reality (VR) technologies have demanded higher standards for multifunctional integration and polarization/wavelength sensitivity. These developments facilitate the seamless connection between free-space and on-chip platforms [11], [12], [13], [14], [15]. Metasurface, a 2D metamaterial, has drawn much attention for its ultracompact footprint, versatile functionalities, and capability of light manipulation at the subwavelength scale [16], [17], [18], [19]. Furthermore, metasurface-integrated waveguides, known as meta-waveguides, present an intriguing configuration for the connection of free-space and photonics platforms, enabling a high degree of freedom (DoF) in light manipulation [20], [21], [22], [23], [24]. For example, the plasmonic antenna–integrated waveguide can be employed for wavelength demultiplexing (WDM) and polarization demultiplexing (PDM) optical communications, offering high transmission speeds but low CE [25], [26]. Moreover, the metasurface can be used for high-efficiency coupling from free-space light to surface plasmon polaritons (SPP) [27], [28], [29], [30]. A single plasmonic catenary meta-atom is employed for an ultra-broadband spin-controlled directional router, achieving ER exceeding 15 dB within a 250 nm bandwidth, while the maximum CE remains below 6 % [31]. Then, dielectric metasurface arrays, designed based on Pancharatnam–Berry (PB) [32] and propagation phases [33], are developed to achieve high coupling efficiency for polarization demultiplexing (PDM), wavelength demultiplexing (WDM), and mode-selective directional coupling. The maximum CE is approximately 67 % for linear-polarization incident conditions and 57 % for spin incident conditions. The BW remains constrained to around 100 nm [34], [35], [36]. Furthermore, an inverse-designed free-form silicon meta-coupler is tailored to achieve incident-guided mode polarization decoupling [37]. The maximum CE and BW are still inferior to those of conventional coupling strategies (see Table S1 in Supporting Information Note S1 for detailed comparison). The presuppositions inherent in the conventional unit-cell–based design approach lead to high sensitivity of diffraction angle and efficiency to incident wavelength variations. Consequently, the phase and mode matching condition is disrupted as the angle varies due to wavelength detuning, resulting in the degradation of both CE and BW. Furthermore, for spin incident conditions, the dominant coupled mode is TM mode [36]. The polarization mismatching can further impose limitations on the devices performance. Previous research on free-space meta-devices demonstrate that continuous structures are beneficial for designing high-efficiency and broadband diffraction devices [38], [39]. In particular, as a novel geometry, a catenary-inspired structure can generate continuous phase profiles over a broad spectral band, effectively overcoming adjacent coupling issues [40], [41]. Detailed theory and applications of catenary optics can be found in previous works [42], [43]. While the single catenary meta-atom integrated waveguide coupler has demonstrated potential for spin-demultiplexing across a wide range of bandwidth, its efficiency still needs enhancement [31]. Therefore, overcoming the limitations of CE and BW for spin-selected meta-waveguide couplers is an urgent problem to be addressed.

**Figure 1:**
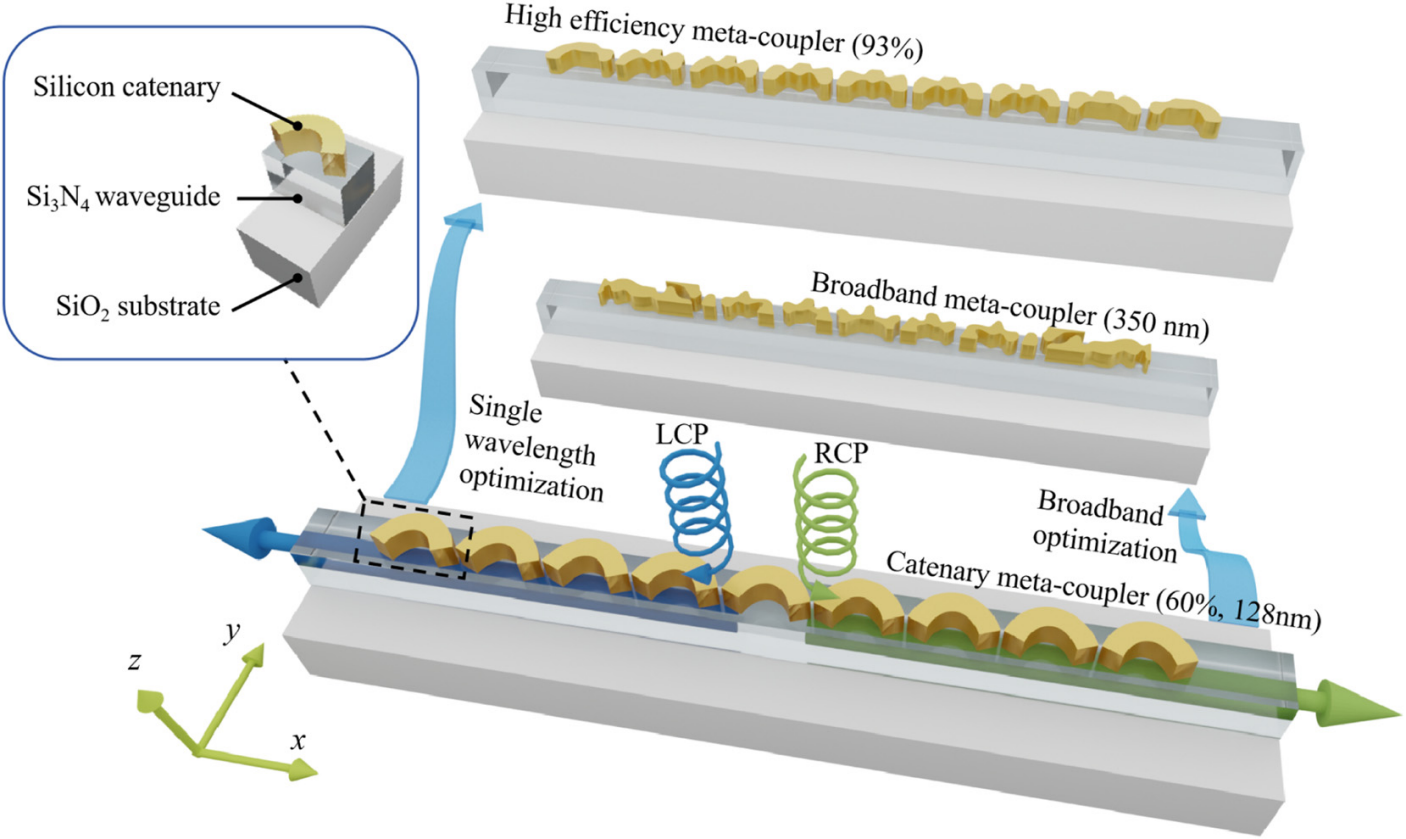
Vertically incident circularly polarized light can be coupled unidirectionally into a specific output port using an integrated metasurface consisting of nine silicon catenary structures. High coupling efficiency and broad bandwidth free-form meta-couplers can be achieved based on single-wavelength and broadband optimization strategies, respectively.

In this work, effective index curves and far-field radiation distribution are derived based on diffraction analysis. The results indicate that spurious diffraction orders and near-field destructive interference are the main reasons for limited performance in both discrete and quasi-continuous conditions. The utilization of the catenary array efficiently increases the CE while it still suffers from low BW and ER. To address the limitations of the aforementioned meta-couplers, an adjoint-based topology optimization method is utilized for near-field customization, as shown in Figure 1. The adjoint-based algorithm can iteratively enhance the similarity between real electric fields to an ideal distribution within the design region [44]. The spurious diffraction orders and the destructive interference caused by adjacent coupling are effectively suppressed by free-form metasurface, achieving a maximum CE of over 90 %. Furthermore, using a multiobject optimization method, we have demonstrated a broadband spin-selective meta-coupler with a BW exceeding 350 nm, covering the O- to L-bands. The average CE for the entire band is around 50 %, with a maximum CE of 60 %. This optimization method may provide a new design strategy for other integrated meta-devices, including on-chip holography generators, biosensors, and optical orbital angular momentum generators.

## 2 Forward designed meta-couplers with limited performance

### 2.1 Discrete and quasi-continuous meta-couplers

**Figure 2:**
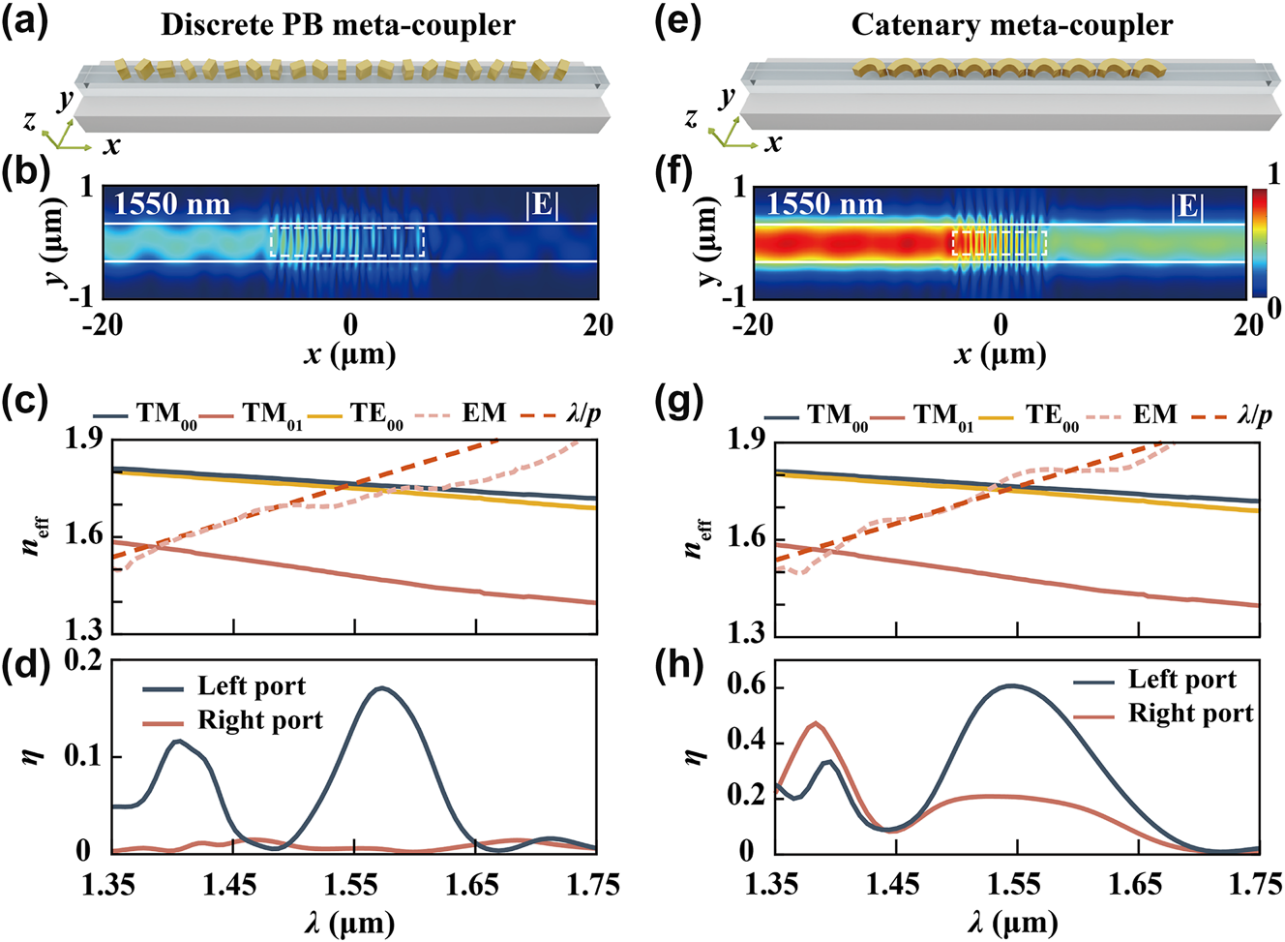
Forward-designed meta-couplers. (a) and (e) Schematics of meta-couplers designed using discrete PB phase meta-atoms and truncated catenary structures; (b) and (f) guided mode distributions within the waveguides under left circular polarization incident condition; (c) and (g) estimated *n*_eff_ curves of metasurface phase-gradient, excited modes (EM) and eigenmodes, including TM_00_, TE_00_, and TM_01_ modes; (d) and (h) guided mode distributions within the waveguides under left circular polarization incident condition.

Two meta-couplers are designed for performance comparison, one with discrete squared meta-atoms on top and the other with catenary meta-atoms integrated. The size of the catenary is mainly determined by the waveguide width and phase-matching condition. Therefore, the truncated equal strength catenary structure is used here for the size fitness between the waveguide and meta-atom (Supporting Information Note S2 and S3). We have demonstrated the CE of the catenary-integrated metasurface does not continue to increase with the number of catenary structures. In addition, the ER exhibits a reduction as the meta-atom number increases due to the spurious diffraction orders (Supporting Information Note S4). A catenary array containing nine meta-atoms is used as an initial structure for comparison here and optimization further. Figure 2 illustrates two meta-gratings with same phase gradient designed for TM_00_ mode-selective unidirectional coupling at 1,550 nm. The performance of two meta-couplers under left circular polarized light illumination is numerically verified for comparison. Figure 2b and f illustrate the guided mode distributions within the Si_3_N_4_ waveguides. Unidirectional coupling is demonstrated for both conditions, while the discrete meta-coupler exhibits a lower performance. Figure 2c and g show the phase-matching conditions between the metasurface excited mode (EM) and eigenmodes in a broad bandwidth.

The effective index of EM (*n*_eff|EM_) is estimated using a near-field-based wave-vector analysis schematic (Supporting Information Note S5) [2]. In addition, the effective index corresponding to the metasurface with ideal phase gradient is defined as *n*_eff|ideal_ = *λ*/*p*. In which, *p* is the ideal period with 2*π* phase gradient. For discrete metasurface conditions, several meta-atoms are contained in the ideal period *p*. In the case of catenary metasurfaces, single catenary structure is contained in *p*. The cross-points between the eigenmodes effective index *n*_eff_ curves and EM effective index *n*_eff|EM_ curves indicate the perfect wave-vector matching between EM and eigenmodes. Beyond these cross-points in wavelength, the coupling mechanism relies on quasi-matching conditions, which lead to a reduction in efficiency [35], [45]. Similarly, the cross-points between *n*_eff| ideal_ and *n*_eff_ curves indicate the ideal wave-vector matching conditions. However, the *n*_eff|ideal_ can be influenced by several factors, including adjacent meta-atoms coupling, metasurface dispersion, and near-field interference caused by waveguide constraints, leading to a nonlinear feature of *n*_eff| EM_ curves. The *n*_eff| EM_ obtained from full-wave simulations account for these factors, making it more accurate for evaluating phase matching conditions than using *n*_eff| ideal_. As can be seen, the first (around 1,550 nm) and second (around 1,400 nm) peak positions of the CE spectra shown in Figure 2d and h exhibit a better consistency with the *n*_eff| EM_ cross-points, compared with *n*_eff|ideal_ cross-points.

In Figure 2c, the matching positions between *n*_eff_ and *n*_eff|EM_ for discrete condition exhibit an extended phase-matching region around 1,550 nm. This extension causes the left port CE peak position to shift by 27 nm, from 1,550 nm to 1,573 nm. This phenomenon is challenging to control for the discrete conditions because the meta-atom database is obtained based on single-wavelength sampling. In contrast, for quasi-continuous conditions, the inherent spectrally dispersionless property leads to a better match between the *n*_eff| EM_ and *n*_eff|ideal_ curves [46]. This results in a small drift in the CE peak position of only 3 nm (from 1,550 nm to 1,547 nm). Furthermore, the coupling mode purities at 1,550 nm achieved by discrete PB meta-coupler is 80 % for TM_00_ and 17 % for TE_00_ mode, due to the phase gradient of the discrete metasurface is arranged according to the effective index of the TM_00_ mode (Supporting Information Note 2 and 3). Because of the phase mismatching condition, the *x*-polarized component of the incident circularly polarized beam is difficult to couple efficiently into the TE mode [27], [28]. The polarization mismatching conditions can be addressed by using a propagation phase-based metasurface. Additionally, the CE and the 3 dB BW are lower for the discrete condition than compared to the quasi-continuous condition (13 % < 60 % and 85 nm < 128 nm). The imperfect phase-matching caused by the discrete phase-sampling strategy increases the wavelength sensitivity of the coupling phenomenon [3], [47]. Small detuning of the incident wavelength causes a significant and uncontrollable variation in the diffraction angle, leading to a decrease in BW. It should be noted that, a meta-coupler formed by only 21 meta-atoms arranged in a single row is shown here, and thus, a 13 % circular polarization coupling efficiency is relatively low. However, improving the efficiency of discrete meta-couplers is feasible using only forward design methods. Potential strategies include using propagation phase for linearly polarized light coupling [36], increasing the number and density of meta-atom arrangements [35], [36], or employing wider waveguides for higher-order mode coupling [35].

The high performance for quasi-continuous conditions demonstrates the superiority of continuous structures in achieving high-efficiency diffraction, consistent with previous studies [39], [48], [49]. Additionally, the satisfaction of the phase-matching condition indicates the linear PB phase relationship Φ(*x*) = 2*σθ*(*x*) holds for the subwavelength-scaled catenary [31]. Here, *θ* and *Φ* represent the tangent angles and the phase response at position *x* on the catenary curve, and *σ* = ±1 represents the chirality of incident light. Furthermore, even though quasi-continuous meta-coupler exhibit higher performance in terms of CE and BW, the ER at 1,550 nm is higher for the discrete condition (13 dB > 4.7 dB).

### 2.2 Coupling efficiency and bandwidth limitations

**Figure 3:**
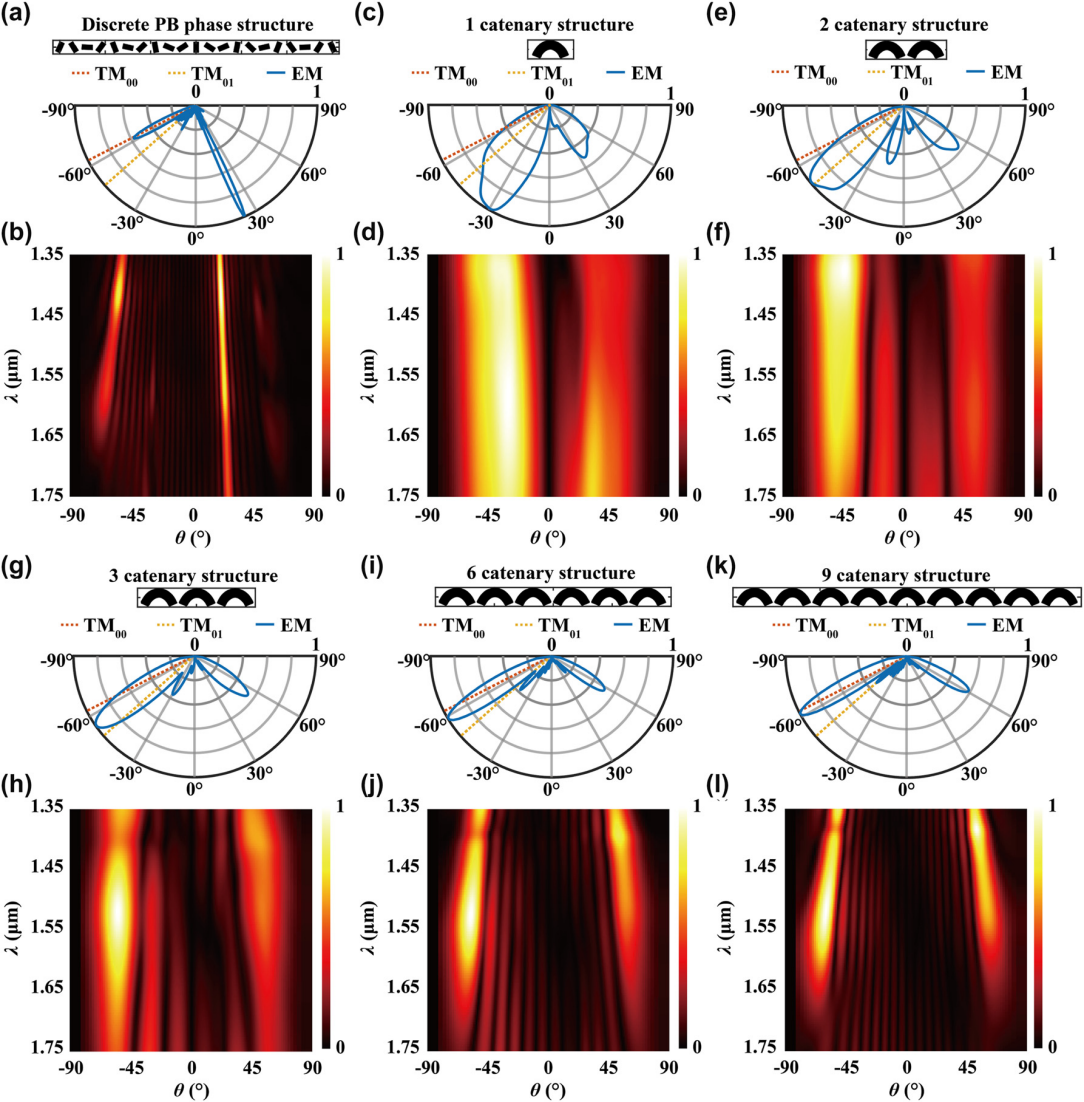
Far-field radiation analysis. (a) Farfield radiation diagram at 1,550 nm for discrete metasurface; (b) diffraction intensity spectrum at far-field for discrete metasurface; (c–l) radiation diagrams at 1,550 nm and intensity spectrum for metasurface with different numbers of catenary structures. The numbers are 1, 2, 3, 6, and 9, respectively. The insets show the metasurface structures, with detailed sizes can be found in Supplementary Note 2.

To understand the performance limitations of the meta-couplers, far-field diffraction analysis is conducted. Figure 3 presents the far-field radiation spectrum of the z-polarized electric fields, with E_*z*_ being the predominant electric component of the TM mode. The radiation diagrams represent the diffraction behaviors specifically at 1,550 nm, while the intensity spectrum represents the diffraction behaviors across a broad bandwidth. This analysis is based on the finite difference time domain (FDTD) method [50], [51]. The diffraction angle of TM_00_ and TM_01_ mode at 1,550 nm is 63° and 48°, respectively (Supporting Information Note S5 and S6). As shown in Figure 3a, even though the phase-matching condition at 1,550 nm for TM_00_ mode in the discrete metasurface is satisfied, a significant portion of the energy is dispersed into spurious diffraction order. The wave vector of the subdiffraction order matches with neither TM_00_ nor TM_01_ mode, leading to a low coupling efficiency to the right port. Suppressing the spurious diffraction order for the discrete phase-sampling condition is challenging due to local period assumptions and adjacent coupling phenomena. Consequently, achieving and maintaining high CE performance for each conventional discrete-phase sampling-based meta-waveguide couplers is difficult. Tedious meta-atom parameter sweeping and arrangement testing procedures are required for each meta-coupler device in previous work [35], [36].

In Figure 3c–l, the far-field distributions of catenary arrays with varying numbers of meta-atoms are illustrated. The radiation diagram of a single catenary shows a broad angle range of approximately 30° toward the left direction at 1,550 nm, as depicted in Figure 3c. Most of the scattered power from single subwavelength catenary is distributed across various diffraction angles instead of efficiently coupling to the TM_00_ or TM_01_ mode. The wide diffraction angle spectrum, shown in Figure 3d, results in a broadband coupling phenomenon while with limited CE. As the number of catenary increases, the diffraction angle range gradually decreases and aligns more closely with the ideal angle represented by the TM_00_ mode. This alignment leads to an increase in coupling efficiency at 1,550 nm owing to the satisfaction of phase-matching condition and reduced power dissipation (Supporting Information Note S4).

Increasing the number of catenary structures does not fully suppress the subdiffraction orders. Additionally, it results in a narrower range of diffraction angles, which enhances alignment with the eigenmode propagating toward the right port. Consequently, the ER decreases with the increasing number of catenary structures (Supporting Information Note S4). In addition, the destructive interference of near-field distributions is observed when the metasurface contains more than six catenary structures (Supporting Information Note S6). As a result, the catenary-array–assisted meta-waveguide coupler exhibits poor directivity and limited coupling efficiency. The primary factors contributing to the CE and BW limitation are the presence of spurious diffraction orders and the coupling phenomenon between adjacent meta-atoms.

## 3 Inverse designed meta-couplers with customized near-field distributions

### 3.1 Adjoint-based optimization for ultra-high coupling efficiency

An adjoint-based topology optimization algorithm is utilized for near-field customization to suppress the spurious order and adjacent coupling phenomenon. Full-model optimization can modulate the mutual coupling effects between adjacent meta-atoms, making them beneficial for unidirectional high-efficiency coupling. Customized near-field can be implemented because free-form structures support many more Bloch modes compared to forward-designed metasurfaces [48], [52]. A vertically incident left-circular polarized plane wave is utilized as the forward simulation source. Target output mode determined sources are utilized in the adjoint simulation as the backward-propagating sources at dual-ports (Supporting Information Note S7). The ideal field distribution beneath the metasurface region can be obtained with adjoint source transmission from these ports. The adjoint-based optimization algorithm can iteratively enhance the similarity between the actual and target field distributions [53], [54]. The figure of merits (FoM) for the dual-port optimization can be expressed as:

(1) $$\begin{array}{ll} {\text{FoM}}_{\text{port}1} & =\iint f_{1}\left(E\left(y,z\right)\right)\;dy\;dz\\ & =\iint \left|E_{\text{port}1}\left(y,z\right)\cdot E_{\text{port}1}^{*}\left(y,z\right)\right|\;dy\;dz \end{array}$$

(2) $$\begin{array}{ll} {\text{FoM}}_{\text{port}2} & =\iint f_{2}\left(E\left(y,z\right)\right)\;dy\;dz\\ & =\iint -\left|E_{\text{port}2}\left(y,z\right)\cdot E_{\text{port}2}^{*}\left(y,z\right)\right|\;dy\;dz \end{array}$$

where E_port1/2_(*y*,*z*) represents the complex electric field of the output mode at the corresponding outport in forward simulation and * represents the complex conjugate operation. This design ensures that the intensity of coupled mode in port1 (which is the left port in this work) is enhanced, while the intensity in port2 (which is the right port in this work) can be efficiently suppressed.

**Figure 4:**
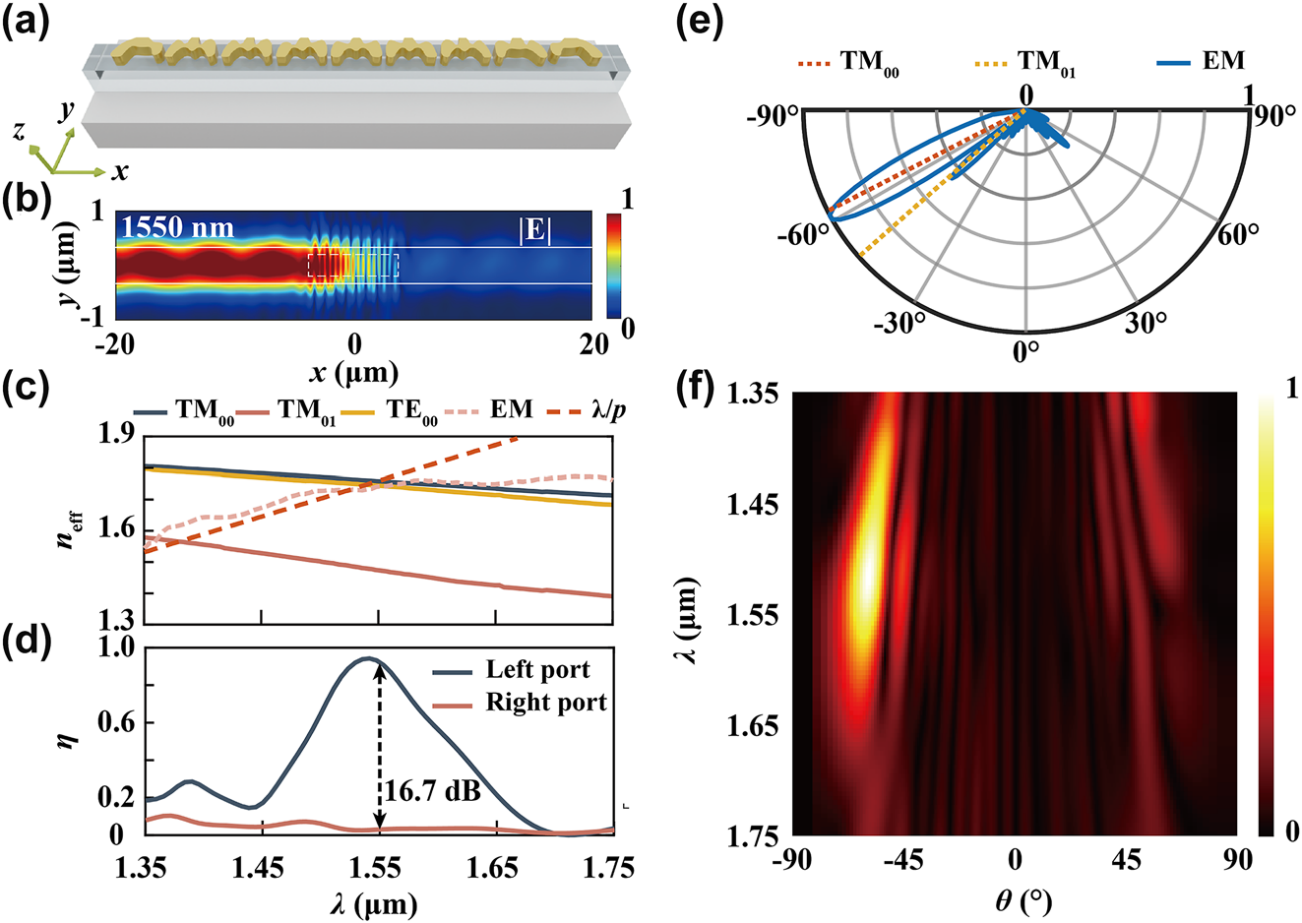
High-efficiency meta-coupler. (a) Schematic of high coupling efficiency (CE) free-form meta-coupler using single-wavelength optimization strategy; (b) guided mode distribution within the waveguide under left circular polarization incident condition; (c–d) coupling efficiency and *n*_eff_ spectra; (e) radiation diagram at 1,550 nm; (f) intensity spectrum for high CE meta-coupler.

The initial structure utilized is the quasi-continuous catenary array with nine meta-atoms, depicted in Figure 2e. The optimized structure is shown in Figure 4a, featuring a free-form catenary-inspired design. Different from the repeated arrangement of identical catenary meta-atoms, this customized metasurface is constructed with different catenary-inspired meta-atoms. It is noticeable that the meta-atoms at the edges of the array are significantly different from those in the central region. This variation can be attributed to the change in adjacent coupling conditions at the ends of the array, due to the absence of the neighboring structures. This indicates that the near-field coupling between adjacent meta-atoms has been effectively optimized to generate a customized field distribution. The E_*z*_ component of near fields exhibits a more accurate diffraction angle and a constructive interference compared to forward design conditions (Supporting Information Note S8). The guided mode field distribution and spectra shown in Figure 4b–d indicate a significant directivity (around 16.7 dB) and ultra-high coupling efficiency (around 93 %) at 1,550 nm. Since the optimization procedure is conducted at a single wavelength, the 3 dB BW remains around 130 nm. In addition, a small blue-shift of the coupling peak position also remained, for the maximum coupling efficiency of 97 % is achieved at 1,540 nm.

The *n*_eff_ curve and far-field radiation shown in Figure 4d and e indicate a perfect phase matching between EM field and TM_00_ mode at 1,550 nm. The mode purity of left port mode at 1,550 nm is calculated as 95 % and 3 % for TM_00_ and TM_01_ mode, respectively. High TM mode purity indicates that the free-form meta-coupler has integrated polarization conversion capability [37]. Moreover, the high coupling efficiency of free-form meta-coupler demonstrates that the topology optimization algorithm can effectively address the polarization mismatch issue in the circular polarization coupling conditions. The far-field radiation spectrum in Figure 4f shows that the maximum intensity is around 1,550 nm, which is consistent with the coupling efficiency spectrum. Additionally, the spurious diffraction order is well suppressed at 1,550 nm owing to the dual-port optimization strategy.

### 3.2 Adjoint-based optimization for broadband coupling

To address the bandwidth limitation of the diffraction based meta-coupler, a multiobjective topology optimization strategy is employed to design a high-efficiency broadband meta-waveguide coupler over the O-, S-, C-, and L-bands. The goal is to maximize the average coupling efficiency within these bands at the target port1. The figure of merit (FoM) for this objective can be expressed as:

(3) $$\begin{array}{ll} {\text{FoM}}_{\text{max}-\text{min}} & ={max-min}_{i\in \left\{1,2,\ldots ,N\right\}}CE\left(\lambda_{i},\text{port}1\right)\\ & =\iint \left|E_{\lambda_{\min},\text{port}1}\cdot E_{\lambda_{\min},\text{port}1}^{*}\right|\;dy\;dz \end{array}$$

In which, the **CE**(*λ*_*i*_, port1) denotes the coupling efficiency at the port1 and wavelength *λ*_*i*_, while the **max-min** signifies maximizing the minimum coupling efficiency within the target band at *λ*_min_.

**Figure 5:**
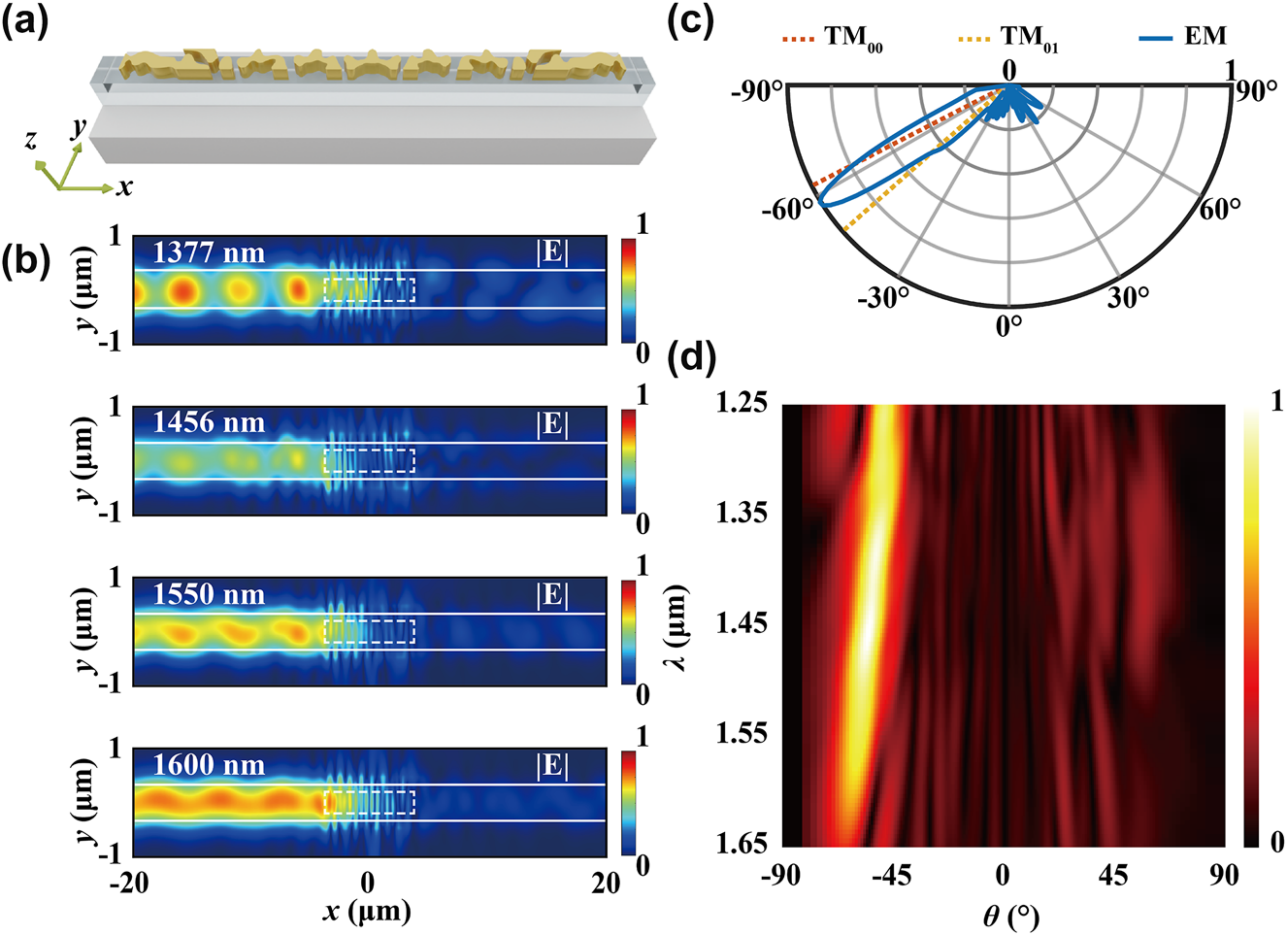
Broadband meta-coupler. (a) Schematic of broad bandwidth (BW) free-form meta-coupler using single-wavelength optimization strategy; (b) guided mode distributions within the waveguide at different wavelengths under left circular polarization incident condition; (c) radiation diagram at 1,550 nm; (d) intensity spectrum for broad BW meta-coupler.

The adjoint source of *λ*_min_ at port2 is determined by Equation (2) to suppress the coupling efficiently in port2, thereby achieving high ER. During the multiobjective optimization procedure, time-domain simulation is employed as the forward process to efficiently obtain the forward field distributions and CE spectrum in the target band. Consequently, the gradient at *λ*_min_ can be selectively derived according to the coupling performance within the designed band (Supporting Information Note S7). The optimized broadband free-form catenary-inspired meta-waveguide coupler is represented in Figure 5a featuring symmetric free-form catenary-inspired structures. It is worth noting that the continuous feature is more pronounced for broadband coupling conditions. The last two catenary meat-atoms at the edges of the array are replaced by a single continuous free-form structure. These structures play a crucial role in generating customized near-fields with specific diffraction angles and uniform efficiency at different wavelengths (Supporting Information Note S9). The coupling mode distributions within the waveguide at four different wavelengths are shown in Figure 5b. These wavelengths are selected based on the mode purity spectrum, specifically targeting maximum purity for TM_01_ at 1,377 nm and TM_00_ at 1,600 nm, respectively. Additionally, the hybrid mode at 1,456 nm plays a critical role in revealing the mechanism behind bandwidth expansion. As can be seen, spin-selective unidirectional coupling is realized at these wavelengths with significant ERs. The radiation diagram at 1,550 nm, shown in Figure 5c, indicates a quasi-matching condition based on hybrid mode coupling, as the far-field radiation angle exits between the TM_00_ and TM_01_ modes. In addition, a uniform diffraction intensity distribution and suppressed spurious diffraction orders over the target band are achieved thanks to optimized free-form structures, shown in Figure 5d.

**Figure 6:**
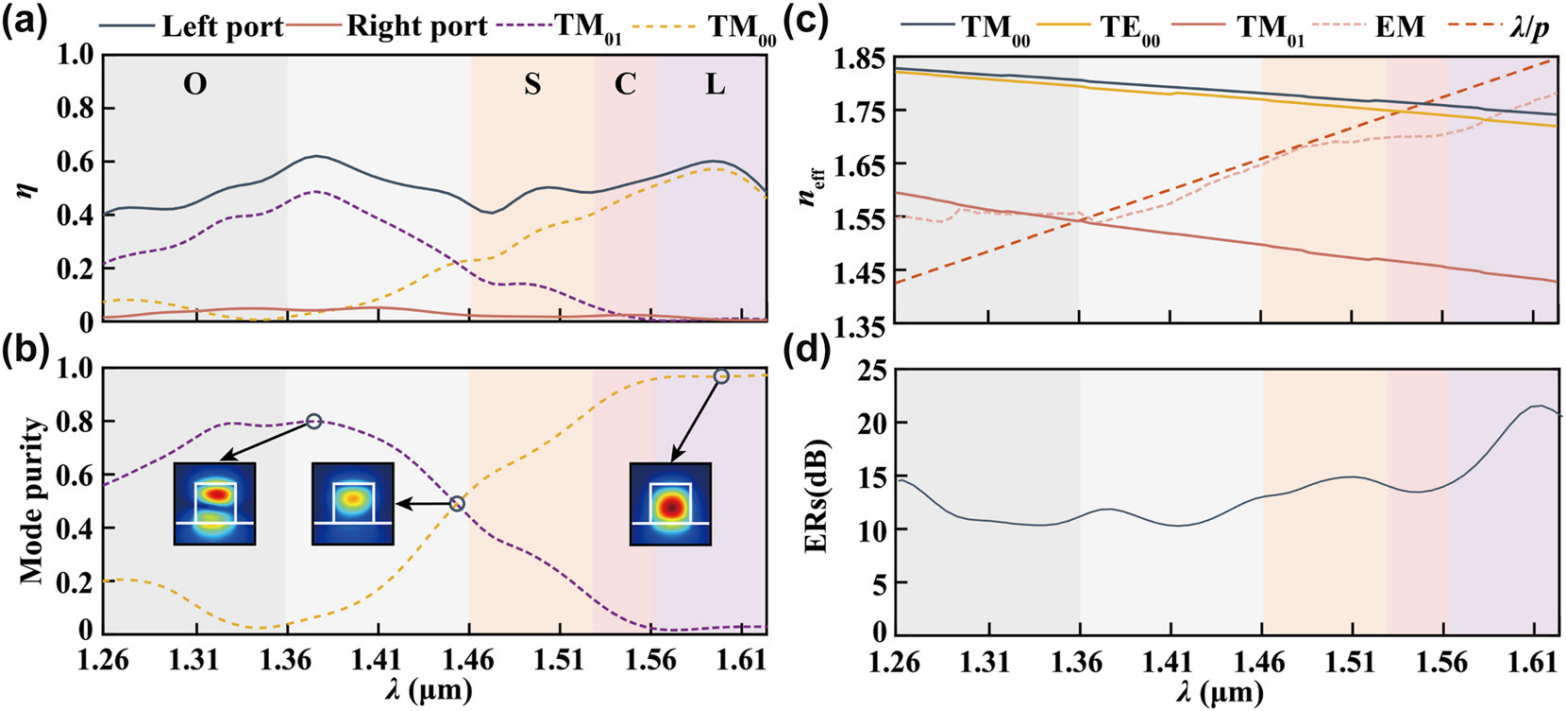
Spectra demonstration of the broadband meta-coupler. (a) Coupling efficiency spectra cover the O-, S-, C-, and L-band. The dominant mode is mainly TM_01_ in O-band, TM_00_ in C- and L-band, and hybrid mode in S-band; (b) the mode purity spectra of TM_00_ and TM_01_ modes. The insets represent the left port mode distributions at 1,377, 1,456, and 1,600 nm, respectively. The white line represents the cross section of waveguide; (c) effective index *n*_eff_ spectra; (d) extinction ratios (ERs) spectra. The minimum ER in the target band exceeds 10 dB and the average ERs is 13 dB.

The average CE of the left port in O- to L-bands is over 50 %, as shown in Figure 6a. To reveal the broadband coupling mechanism, the mode expansion method is utilized to calculate the mode distribution over the target band [36]. Mode purity is defined as the ratio of eigenmode power and total output power at the left port. The maximum TM_00_ and TM_01_ mode purities of output mode are 80 % and 96 %, respectively, as shown in Figure 6b. The lower mode purity of the TM_01_ mode results from the fact that the primary optimization target in this work is coupling strength. Since the target mode is not defined in the FOM, the optimization algorithm may select multiple eigenmodes as the target modes in each iteration. Therefore, the mode purity can be further enhanced by adding the mode similarity objective into the FoM [37]. Alternatively, efficiency and purity optimization can be performed separately, as different objective functions will prevent convergence issues. The corresponding mode distributions at the left port are illustrated as the insets within the coupling spectrum. The output mode gradually changed from TM_01_ at 1,377 nm to TM_00_ at 1,600 nm. The broadband optimization algorithm can gradually modify the near-field distribution at the least efficient positions within the operating bandwidth, identifying and coupling to corresponding eigenmodes (Supporting Information Note S9). For each mode coupling, the bandwidth is enhanced based on the customized phase-matching conditions shown in Figure 6c. In this figure, the extended aligned region at 1,377 nm and 1,600 nm, combined with uniform diffraction intensity, leads to a broadband mode-selective coupling. The effective index curve *n*_eff|EM_ exhibits a significant difference from the ideal linear distribution *n*_eff|ideal_ around the aligned position, indicating a different diffraction angle dispersion feature.

Additionally, the TM_00_ peak CE position of the initial catenary meta-coupler is at 1,550 nm, while the broadband optimized meta-coupler represents a TM_00_ peak position at 1,600 nm. The significant red shift in the coupling peak is attributed to the customized dispersion induced by the free-form structure. It is worth noting that, different from the uncontrollable dispersion shown in Figure 2c induced by conventional discrete phase-sampling strategies, the nonlinear dispersion here is customized for bandwidth enhancement. Furthermore, with efficiently suppressed CE at the right port, the minimum ER in the target band exceeds 10 dB and the average ER is 13 dB. Meanwhile, the adjoint source at the reverse port continually suppresses the generation of spurious diffraction, which also facilitates the convergence of coupling energy toward the target port. The bandwidth optimization mechanism, therefore, can be attributed to the multimode coupling phenomenon. By modulating the target spectra width, the bandwidth can be easily adjusted. Moreover, the full model topology optimization method can be extended to different waveguide platforms, such as the coupling between two waveguides or between a waveguide and an optical fiber [5], [8], [20], [21]. By designing appropriate figures of merit, gradients for performance enhancement or the implementation of exotic functions can be calculated through forward and adjoint simulations.

## 4 Conclusions

In this paper, the coupling efficiency (CE) and bandwidth (BW) limitations associated with conventional discrete metasurfaces and catenary array–assisted waveguide couplers are comprehensively demonstrated. These limitations are dominantly induced by destructive near-field interference and spurious diffraction orders. Meta-couplers constructed with quasi-continuous catenary structures exhibit superior CE and BW performance compared to the discrete conditions, attributed to continuous phase responses and reduced mutual coupling between adjacent meta-atoms. However, forward design methods cannot fully overcome the limitations of CE and BW. To address these challenges, an adjoint-based topology optimization algorithm is employed to achieve customized near-field distributions. By adopting dual-port and multiobjective optimization strategies, two free-form meta-couplers with ultra-high coupling efficiency and broad bandwidth are implemented, respectively. The maximum coupling efficiency reaches 93 % with a high ER of 16.7 dB at 1,550 nm. The broad bandwidth spans 350 nm with an average CE of 50 % and an average ER of 13 dB across the O- to L-bands. These results highlight the significant degree of freedom (DoF) in mode manipulation capabilities of free-form chip-integrated metasurfaces. The topology optimization method is readily applicable to other metasurfaces on unconventional facets, such as meta-fibers, source-integrated metasurfaces, and light-emitting metasurfaces [21]. Importantly, the inverse design strategy represents a pivotal step toward exploring and overcoming performance limitations in metasurface-integrated devices.

## Supplementary Material

Supplementary Material Details
