# Supplementary material for: Free-form catenary-inspired meta-couplers for ultra-high or broadband vertical coupling
Source: Nanophotonics. 2025 Jan 8;14(8):1145–55. doi: 10.1515/nanoph-2024-0566 (PMC12019950; doi:10.1515/nanoph-2024-0566)
Supplement: Supplementary file 1 — Supplementary Material Details [file j_nanoph-2024-0566_suppl_001.docx]

*Supporting Information for*

Free-form catenary-inspired meta-couplers for ultra-high efficiency or broadband vertical coupling

Tianqu Chen, Mingfeng Xu, Mingbo Pu*, Xi Tang, Yuhan Zheng, Qingji Zeng, Yuting Xiao, Yingli Ha, Yinghui Guo, Fei Zhang, Nan Chi, Xiangang Luo

**Supplementary Note 1:** **Comparison of on-chip vertical coupler devices.**

**Table S1.** Summary of on-chip vertical coupler devices.

REF: reference; Forw: forward design; Inv: inverse design; Exp: experiment; Sim: simulation; Max-CE: maximum coupling efficiency; BW: bandwidth; PDM: polarization (de)multiplexing

| **REF** | **Structure** | **λ (nm)** | **Design Strategy** | | **Test**  **Method** | **Max-CE** | **3dB-BW** | **PDM** |
| --- | --- | --- | --- | --- | --- | --- | --- | --- |
| [5] | Plasmonic grating and metal buffer deflection layer | 1550 | | Forw. | Exp. | 72 % | 350 nm | No |
| [6] | Silicon grating and anti-deflection layer | 1310 | | Inv. | Sim. | 88 % | 30 nm | No |
| [8] | 3D polymer structure | 1550 | | Forw. | Exp. | 16 % | 970 nm | No |
| [10] | 3D polymer structure | 1550 | | Forw. | Exp. | 80 % | 500 nm | No |
| [25] | Plasmonic meta-atom | 1550 | | Forw. | Exp. | 5.8 % | ~ | No |
| [26] | Plasmonic meta-atom | 1550 | | Forw. | Exp. | 5 % | 50 nm | Yes |
| [31] | Catenary meta-atom | 1550 | | Forw. | Sim. | 6 % | 250 nm | Yes |
| [34] | Silicon metasurface | 1550 | | Forw. | Sim. | 1 % | 40 nm | Yes |
| [35] | Silicon metasurface | 1550 | | Forw. | Sim. | 51.6 % | 70 nm | Yes |
| [36] | Silicon metasurface | 1550 | | Forw. | Sim. | 67 % | 100 nm | Yes |
| **This work** | **High CE metasurface** | **1550** | | **Inv.** | **Sim.** | **93 %** | **100 nm** | **Yes** |
|  | **Broad BW metasurface** | **1550** | | **Inv.** | **Sim.** | **60 %** | **350 nm** | **Yes** |

Table S1 presents recent notable works on on-chip devices, highlighting their ability to couple vertical incident light into guided modes, similar to the function of the free-form meta-waveguide coupler developed in this study. The primary goals of on-chip coupler research are to achieve high efficiency, broad bandwidth, and multifunctional integration. In this work, we demonstrate free-form meta-coupler devices that exhibit superior functionality (PDM) compared to conventional couplers ^[5,6,8,10]^, and better performance (CE and BW) than those of forward-designed meta-couplers ^[25,26,31,34-36]^.

As can be seen from the list, complex structures such as multi-layer devices ^[5,6]^ and 3D polymer structures ^[8,10]^ contribute significantly to enhancing coupling efficiency (CE) and bandwidth (BW). It is noteworthy that in Ref. [5], the vertical incident light is coupled into plasmonic modes which can be strongly confined within the slot metal-insulator-metal (MIM) waveguide. Achieving a comparable conversion from light to plasmonic modes in a CMOS-compatible photonics platform is challenging, resulting in lower CE and BW compared to those reported in Ref. [5]. Furthermore, these conventional couplers can also exhibit polarization insensitivity and large footprints.

Metasurface-assisted waveguide couplers present a promising solution for achieving high efficiency and polarization-selective coupling. As shown in the table, initial works utilized single plasmonic meta-atoms for wavelength ^[25]^ and polarization (de)multiplexing ^[26]^. To address the low coupling efficiency (CE) and bandwidth (BW) resulting from intrinsic plasmonic absorption, dielectric metasurfaces were introduced. Moreover, the Pancharatnam-Berry (PB) and propagation phase metasurface design strategies have made polarization-selective coupling easier to implement ^[34-36]^. However, the conventional unit-cell-based design approach imposes limitations, restricting the CE and BW of traditional discrete metasurface-assisted couplers to approximately 60% and 100 nm, respectively.

In this paper, we optimize the adjacent coupling phenomenon in forward-designed metasurfaces using a quasi-continuous catenary metasurface as the initial structure and an adjoint-based optimization algorithm as the inverse design strategy. Two meta-couplers with ultra-high CE of 93% and broadband BW of 350 nm are demonstrated. The free-form metasurface-assisted waveguide coupler achieves performance comparable to previous devices with multi-layer and complex structures while preserving polarization sensitivity and maintaining a simple structure. Additionally, the adjoint-based algorithm can be easily extended to design wavelength (de)multiplexing couplers.

**Supplementary Note 2: Forward design procedure for discrete and continuous meta-atoms.**


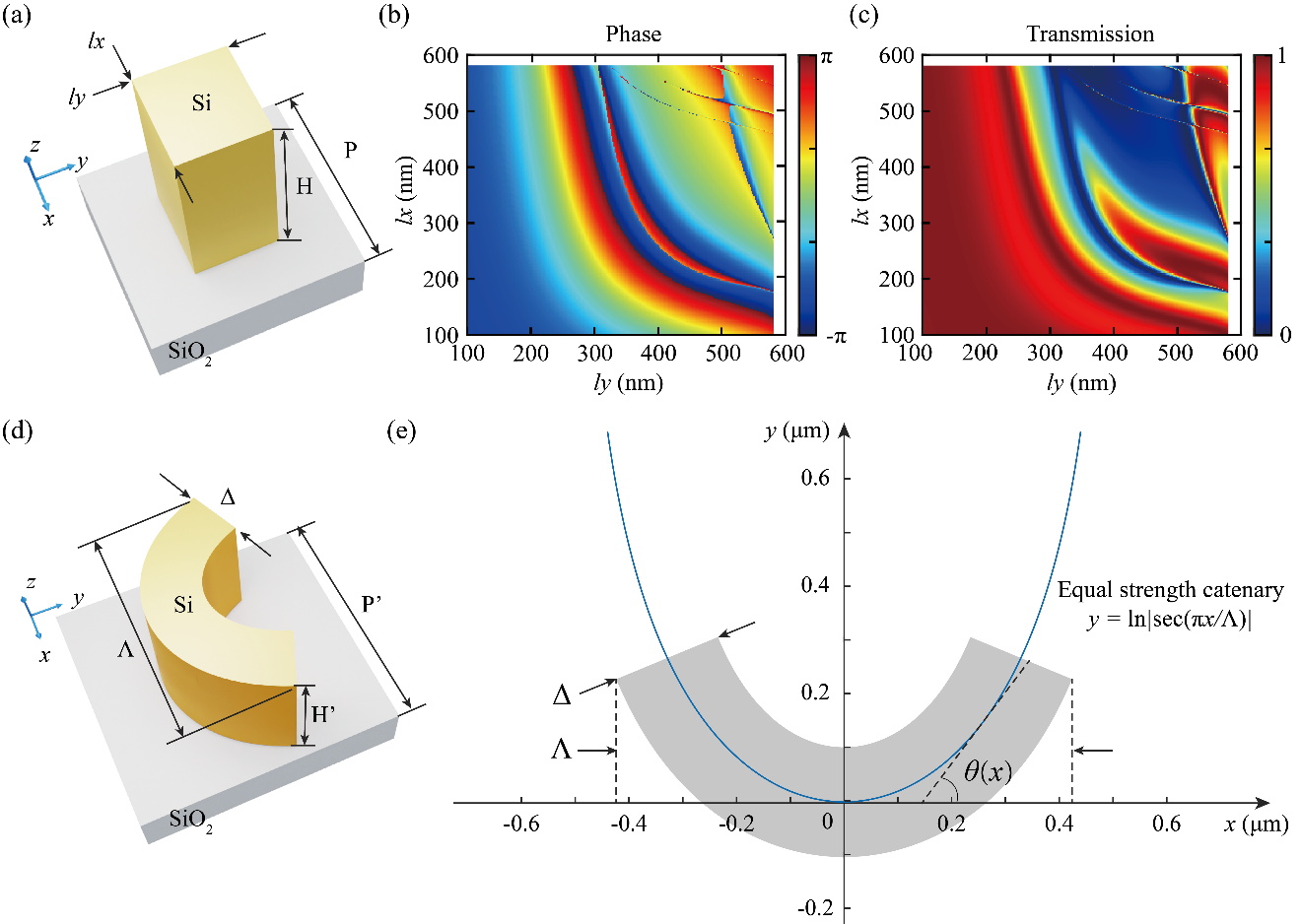


**Figure S1.** (a) Forward designed square Si meta-atom with fixed height H and period P as 1.2 $\text{μm}$and 0.6 $\text{μm}$, respectively; (b-c) Phase and transmission library of unit-cell obtained using RCWA simulation method under y-polarized light incident condition; (d) Forward designed catenary Si meta-atom with fixed height H’ and period P’ as 1 $\text{μm}$and 878 nm, respectively; (e) The trajectory of equal strength catenary and truncated catenary meta-atom topology. The $\theta(x)$represents the tangent angle of the curve at position *x*.

To reveal the limitation of coupling efficiency and bandwidth, a waveguide coupler based on conventional discrete meta-grating is designed. The square silicon meta-atom structure is shown in Figure S1(a). The transmitted phase and transmission library of Si unit-cell (arranged on SiO_2_ substrate) is simulated using rigorous coupled wave analysis (RCWA) solver RETICOLO, shown in **Figure S1 (b-c)**. Detailed description of discrete meta-coupler design procedures can be found in Ref. [36, 37].

Moreover, a truncated equal strength silicon catenary meta-atom structure is also used to compose a quasi-continuous meta-coupler. The structural morphology and the equal strength catenary curve are shown in Figure S1(d) and (e), respectively. Detailed phase response of catenary structure can be found in Ref. [41-43]. Based on the phase matching condition, the transverse wave vector provided by the metasurface needs to be consistent with the propagation vector of the target eigenmode, which can be expressed as:

In which, the is the phase step in the *x*-axis over a distance $\partial d$, the P’ is the catenary grating period, *n*_eff_ is the effective index of the target eigenmode, and *k*_0_ is the wave-vector in the vacuum. Within the waveguide with the size of $1\text{μm}\cdot1\text{μm}$, TM_00_ mode with $n_{\text{eff}\left|\text{TM}_{00}\right.}=1.767$is chosen to be the target mode. For the 1550 nm wavelength incident condition, the period P’ thus can be calculated as 878 nm. The total length $\Lambda$of the catenary meta-atom is set as 846 nm to avoid the overlapping between adjacent structures. The width $\Delta$ is 200 nm for the adaption to the fabrication feature size and the height H’ is 1 $\text{μm}$. The discrete metasurface shown in Figure 2(a) contains 21 meta-atoms and the meta-coupler in Figure 2(e) includes 9 catenary structures. Larger meta-atoms number is selected for the low coupling efficiency will occur under insufficient phase sampling condition. Detailed discrete meta-coupler design strategy is discussed in Supplementary Note 3.


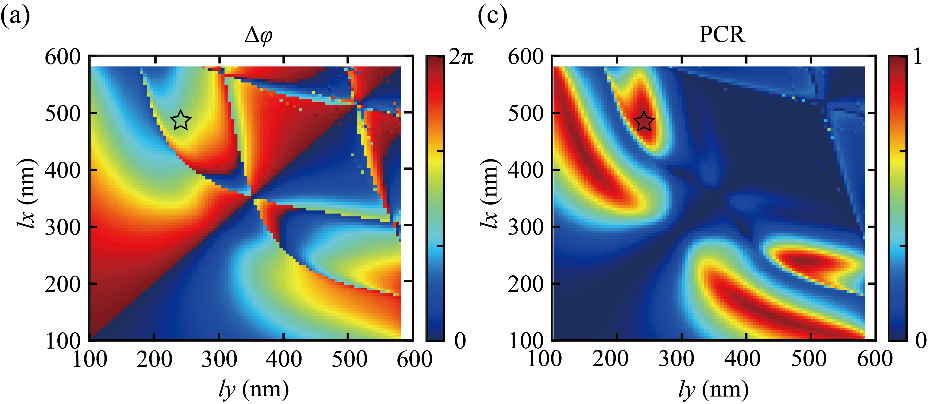


**Figure S2.** (a) Phase difference spectrum () under the condition of *x*-polarized light incidence; (b) The polarization conversion ratio (PCR) spectrum. The positions marked with asterisks correspond to the selected sizes (*l_x_* = 485 nm and *l_y_* = 240 nm) of the discrete PB meta-atom in the text.

According to the principle of Pancharatnam-Berry phase, Jones-matrix of the output electric field can be described as: ^[36]^

$$\left[\begin{matrix} E_{xout} \\ E_{yout} \end{matrix}\right]=\frac{\sqrt{2}}{2}\left((t_{xx}+t_{yy})\left[\begin{matrix} 1 \\ i\sigma\end{matrix}\right]+(t_{xx}-t_{yy})e^{2i\sigma\theta}\left[\begin{matrix} 1 \\ -i\sigma\end{matrix}\right]\right)$$

Therefore, the polarization conversion ratio (PCR) can be defined as: ^[27, 29]^

Figure S2 (a) and (b) illustrate the relationship between the propagation phase difference spectrum and PCR. As can be seen, when the propagation phase difference satisfies , the polarization conversion ratio reaches maximum. Therefore, we choose the size of meta-atom used for discrete meta-coupler shown in Figure 3(a) as *l_x_* = 485 nm and *l_y_* = 240 nm, as indicated by the positions marked with asterisks in Figure S2. The theoretical PCR of this size reaches 97%. However, since the phase gradient of the discrete metasurface is arranged according to the effective index of the TM_00_ mode (Supplementary Note 3), the *x*-polarized component of the incident circularly polarized beam is difficult to be coupled into the TE mode. Due to phase mismatching condition, the coupling mode purities at 1550 nm achieved by discrete PB meta-coupler shown in Figure 2(a) is 80% for TM_00_ and 17% for TE_00_ mode. The near field excited by the circularly polarized light illuminated dielectric metasurface is dominantly coupled to the TM modes within the waveguide. However, the topology optimization method can be utilized to break this limitation by integrating polarization conversion and light coupling functions into the same free-form metasurface structure. ^[37]^

**Supplementary Note 3: Topological morphology of freeform structures.**


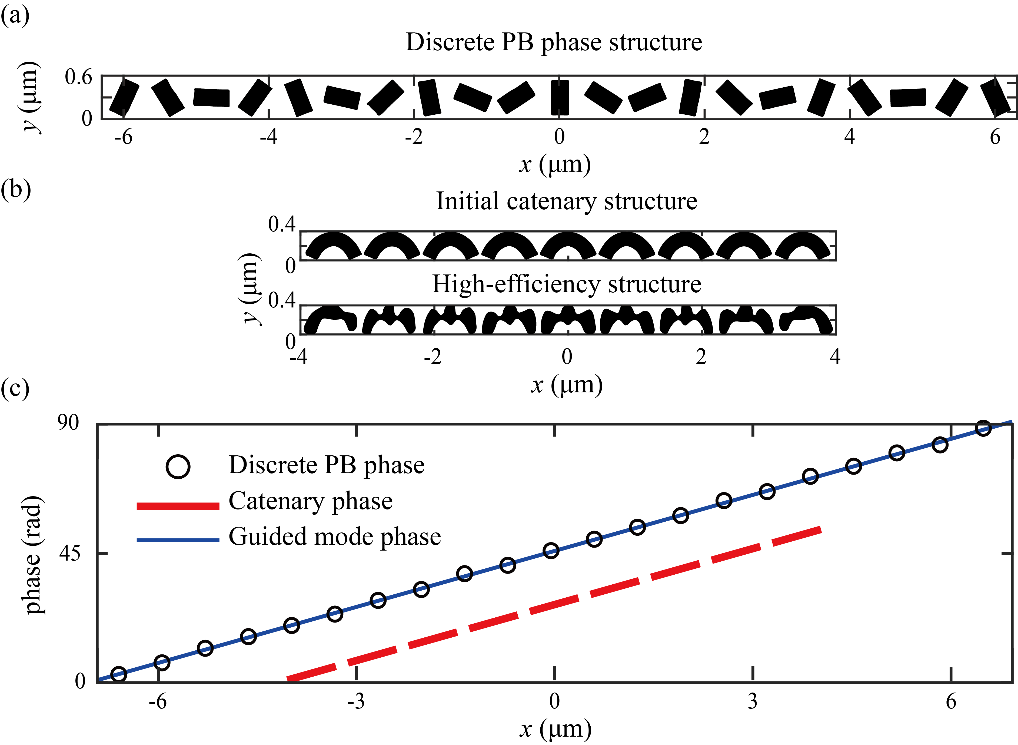


**Figure S3.** Metasurface designed based on phase matching conditions. (a) Discrete PB metasurface; (b) Schematics of the initial catenary array and optimized structures. It consists of freeform amorphous silicon (a-Si) nanostructures patterned on top of a Si_3_N_4_ edge waveguide; (c) Phase matching conditions between target phase gradient (guided mode phase) and metasurface phase gradients.

The phase gradient of target guided mode TM_00_ mode with $n_{\text{eff}\left|\text{TM}_{00}\right.}=1.767$($n_{\text{eff}\left|\text{TE}_{00}\right.}=1.753$) can be obtained based on wave vector transmission theory . For discrete metasurface condition, the period P and the number of meta-atom is 600 nm and $N_{dis}=21$, respectivly. Thus the over a transmission distance $d=N_{dis}\cdot P=12.7\text{μm}$, as shown in Figure S3 (c). Moreover, the phase gradient of the initial catenary structure can also be obtained and shown as the dashed line within Figure S3 (c). Due to the characteristics of the catenary structure, the phase gradient of single catenary structure varies linearly from 0 to 2π within the period within its period $P'=878\text{nm}$. To prevent direct structural overlap of the catenary structure, total length $\Lambda$of the catenary meta-atom is smaller than $P'$leading to a quasi-continuous phase gradient. As can be seen, 9 catenary structures offer a better phase matching condition than 21 discrete PB meta-atoms. Higher coupling efficiency also indirectly confirms the superiority of the quasi-continuous structure.

The optimized coupler devices for high-efficiency coupling and broadband coupling are demonstrated in Figure S3. To enhance the coupling efficiency at a single wavelength, the catenary meta-atoms at various positions are inverse-designed to exhibit distinct topological morphologies, thereby achieving a highly customized electric field. In contrast to the case where most features of the independent catenary structure are preserved under single-wavelength conditions, the structures optimized under broadband coupling conditions exhibit more continuous structural characteristics. This phenomenon further confirms that the performance limitations of the meta-coupler devices arise from the adjacent coupling and the discrete phase sampling. The minimum feature size within the optimized structures is around 60 nm, which can be achieved using CMOS-compatible lithography techniques. ^[20,21]^ This paper primarily discusses the validation and resolution of the reasons behind the poor performance of metasurface integrated coupling devices through inverse design algorithms. Further manipulation of the robustness and the minimum feature size of the designed structures can be achieved through algorithmic approaches. Detailed information about geometric constraints algorithms can be found in our previous works. ^[37, 53]^

**Supplementary Note 4: The coupling efficiency and excitation ratios are influenced by the number of catenary meta-atoms.**

The relationship between CE and ERs has been studied based on the numerical simulations. Figure S4(a) shows that the CE increases with the number of meta-atoms at first, and then is limited to around 60%. ER decreases first and then is stable at 5.5 dB. These phenomena are consistent with the analysis of the near-field above. As illustrated in Figure S4, the catenary number increasing leads to the diffraction angle variation and the emergence of spurious diffraction order. Furthermore, the coupling peak position shifts from 1500 nm to around 1575 nm when the number is lower than 11, and then remains stable around 1575 nm. The slight shift form designed at 1550 nm is induced by the imperfect phase gradient of truncated catenary arrays. Therefore, the catenary array with nine meta-atoms is chosen as the initial structure for optimization over this work.


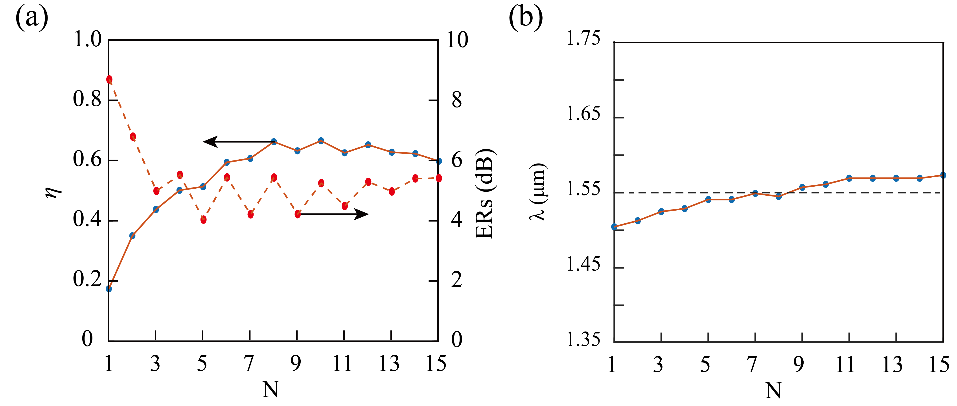


**Figure S4.** (a) Relationships between coupling efficiency $\eta$ at the left port, excitation ratios ERs and catenary numbers; (b) Peak position of coupling spectrum for different catenary conditions. N represent the number of catenary meta-atoms within the metasurface.

**Supplementary Note 5: Near-field distribution of metasurface excited modes.**


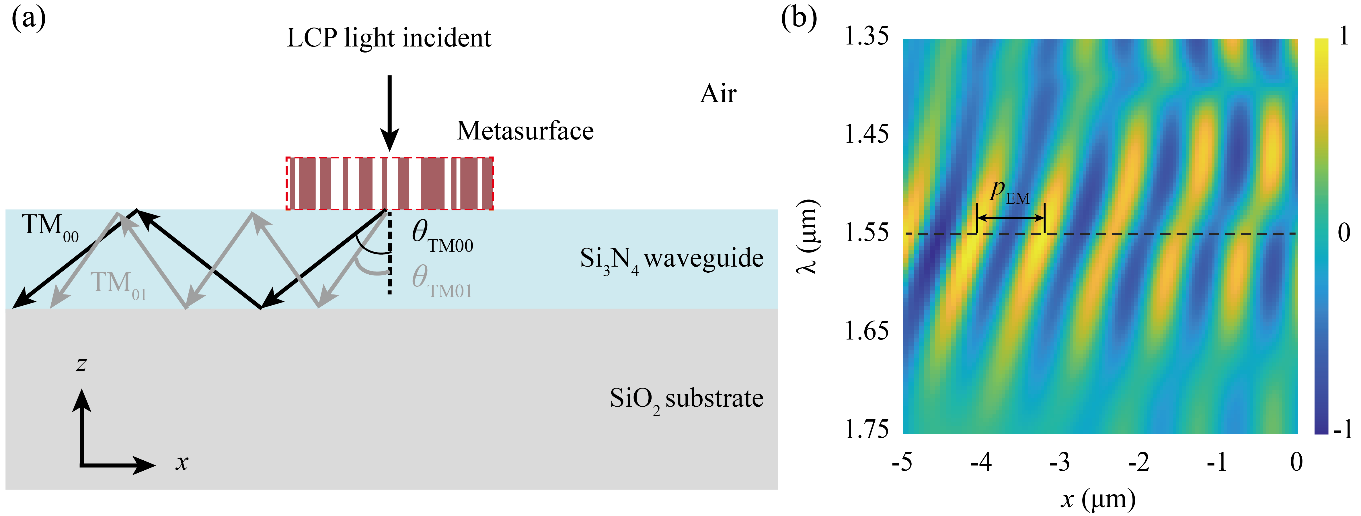
­­­­

**Figure S5.** (a) The metasurface excited modes (EM) with near-field distributions corresponding to different diffraction angles *θ* can be selectively coupled into specific eigenmodes. (b) Near-field E*_z_* distribution spectrum for nine catenary conditions. In which, *p*_EM_ represents the near-field electromagnetic oscillation period.­­­­

The near-field distribution spectrum for nine catenary conditions is obtained using schematics mentioned in Note 5 to avoid near-field interference caused by waveguide constraints. The monitor is set at position 1$\text{μm}$ beneath the metasurface. Based on the wave-vector analysis, the effective index $n_{\text{eff|EM}}$of metasurface excited mode (EM) can be expressed as:

$$n_{\text{eff}|\text{EM}}\cdot k_{0}\cdot p_{EM}=2\pi$$

In which, *p*_EM_ represents the near-field electromagnetic oscillation period. Furthermore, based on the definition of effective index:

$$n_{\text{eff}}\equiv n_{\text{wg}}\sin\theta$$

In which, *n*_wg_ indicates the refractive indices of the waveguide medium and *n*_eff_ is the effective index of eigenmode. The ideal diffraction angle $\theta$ in the waveguide material (Si_3_N_4_) corresponding to a specific eigenmode can be derived from the *n*_eff_ value for the normal incident condition. Therefore, the corresponding diffraction angles *θ* of TM_00_ and TM_01_ modes at 1550 nm are $\theta_{\text{TM00}}=63^{\circ}$ and $\theta_{\text{TM01}}=48^{\circ}$ degrees, respectively. Noticing, the diffraction angle is also suitable for far-field analysis using schematics mentioned in Supplementary Note 6.

In Figure S5 (b), the curve along the dashed line illustrates the near-field distribution along the *x*-axis for the 1550 nm condition. The period for each wavelength can be obtained. Therefore, the effective index curve can be calculated, as shown in Figure 2(c) and (g). Instead of calculating *n*_eff_ based on the phase gradient, the near-field distribution can offer more accurate information of metasurface coupled guide mode, owing to various near-field coupling behaviors considered. The non-linear *n*_eff_ curves shown in Figure 2(c) indicate the diffraction behavior deviates from the intended situations even though the metasurface grating is designed with a linear phase gradient. The non-linear phenomenon can be attributed to the presuppositions hidden in the conventional unit-cell-based design approach, including discrete phase sampling, dispersion less approximation, and local periodicity approximation.

**Supplementary Note 6: Near-field distribution for diffraction analysis**

The near-field distribution is critical for the revealment of the CE and BW limitation. To avoid the interference induced by the bottom reflection of the waveguide, the *z*-axis height of the waveguide is extended from 1 $\text{μm}$to 7 $\text{μm}$. Noticing, the width of the Si_3_N_4_ waveguide remains as 1$\text{μm}$. The schematic of the simulation model with absorbing boundary condition is shown in Figure S6(a).

The E*z* near-field distribution for catenary couplers with different meta-atom numbers is shown in Figure S6(b-f). As can be seen, the spurious diffraction order appears as the number increases, while the diffraction angle range decreases. Furthermore, the destructive interference induced by the adjacent coupling can be seen in Figure S6(d-f). The near-field excited by the meta-atom on the left side is localized beneath the metasurface and contributes little to the unidirectional coupling to the left. Coupling efficiency is therefore limited and cannot be further increased by increasing the catenary number.


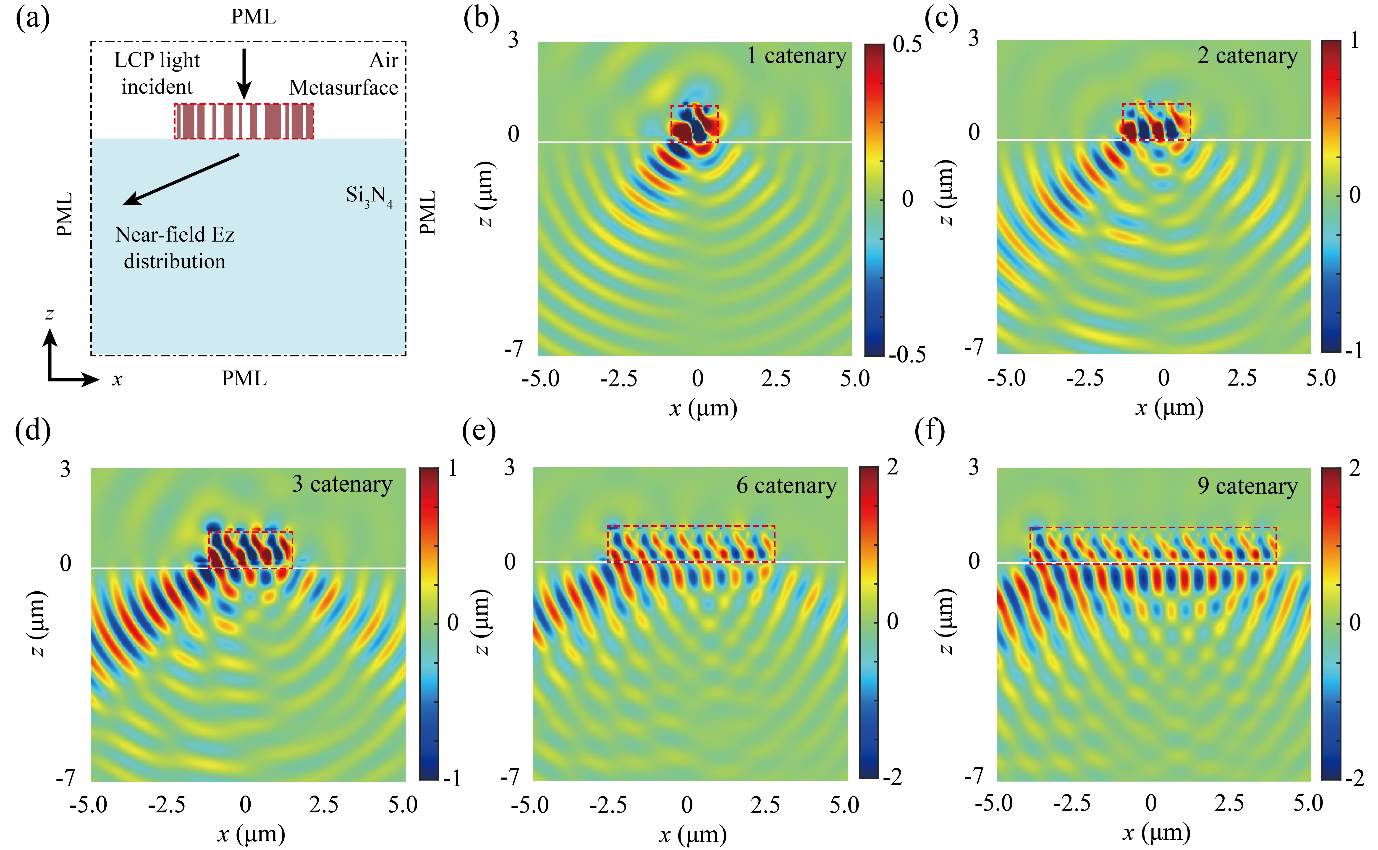


**Figure S6.** (a) Height-extended Si_3_N_4_ waveguide coupler simulation for near-field distribution observation. PML represents the Perfect Matched Layer; (b-f) Z-polarized near-field distributions for catenary array structures with different numbers. The white line and black dashed line represent the air-waveguide interface and metasurface region, respectively.

**Supplementary Note 7: Dual-ports adjoint-based topology optimization strategy for high efficiency and high excitation ratio meta-coupler.**

The figure of merit shown in the main text is designed for the improvement of CE and ERs. The mode intensity at the target port, which is the left port, is the main optimization object. Moreover, the spurious diffraction orders at the right port can be efficiently suppressed by applying constraints at the opposite port. The schematic of the dual-port adjoint-based topology optimization strategy is shown in Figure S7.

Finite-difference time-domain (FDTD) technique was used to perform the full-wave numerical simulations, including the amplitude distributions and the mode profiles. Free-form silicon nano-structure with a fixed height of 1 μm was sitting on a Si_3_N_4_ waveguide. The index of silicon is extracted from the amorphous silicon of the Palik database and set as 3.47 at the wavelength of 1550 nm. A total-field-scattered-field (TFSF) source is used to simulate the normally incident plane wave on the metasurface. ^[36]^ The field in a volume encompassing the patterned metasurface, waveguide, and substrate was simulated and terminated on all sides by perfectly matched layer (PML) boundary conditions.


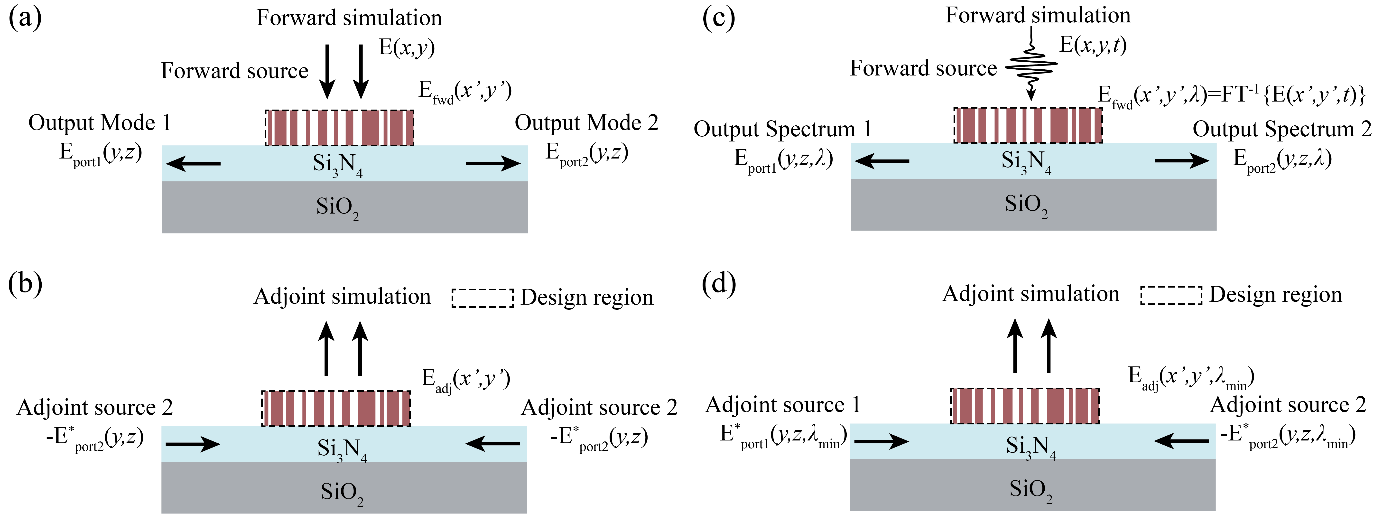


**Figure S7.** (a-b) Schematic of dual-ports adjoint simulation for single wavelength optimization condition; (c-d) Schematic of dual-ports adjoint simulation for broadband optimization condition. E_fwd_ and E_adj_ represent forward and adjoint field, respectively.

For the single wavelength optimization condition, we used the intensity of the electric field at 1550 nm transmitted through the target port as the FOM:

$$\text{FOM}_{\text{port1}}=\iint f_{1}(\text{E}(y,z))\text{d}y\text{d}z=\iint\left|\text{E}_{\text{port1}}(y,z)\cdot\text{E}_{\text{port1}}^{*}(y,z)\right|\text{d}y\text{d}z$$

The FOM for the opposite port is determined as the minus of output mode intensity:

$$\text{FOM}_{\text{port2}}=\iint f_{2}(\text{E}(y,z))\text{d}y\text{d}z=\iint-\left|\text{E}_{\text{port2}}(y,z)\cdot\text{E}_{\text{port2}}^{*}(y,z)\right|\text{d}y\text{d}z$$

where $\text{E}_{\text{port1/2}}(y,z)$represents the complex electric field of the output mode at the corresponding outport in forward simulation and $\mathbf{*}$ represents the complex conjugate operation. The wavelength symbol *λ* is omitted for the wavelength is fixed as 1550 nm. Adjoint simulation is implemented by inversely coupling two adjoint sources into the waveguide from the corresponding outports, offering the desired guided mode distribution under the design region, shown in Figure S7 (a). The two adjoint source distributions are governed by:

$$\frac{\partial f_{1}}{\partial E}=\text{E}_{\text{port1}}^{*}(y,z),\frac{\partial f_{2}}{\partial E}=-\text{E}_{\text{port2}}^{*}(y,z)$$

Moreover, for broadband optimization conditions, the forward source is set as a single pulse with its spectrum covering the target bandwidth from 1250 nm to 1650 nm. By using time domain simulation, the forward field within the metasurface region and the CE spectrum at dual ports can be obtained over the whole bandwidth with single simulation. The figure of merit designed for broadband optimization is expressed as:

$$\text{FoM}_{\max-\min}=\max-\min_{i\in\left\{1,2,...,N\right\}}CE(\lambda_{i},\text{port}1)=\iint\left|E_{\lambda_{\min},\text{port}1}(y,z)\cdot E_{\lambda_{\min},\text{port}1}^{*}(y,z)\right|\text{d}y\text{d}z$$

In which, the $CE(\lambda_{i},\text{port}1)$means the coupling efficiency at the port1 and wavelength $\lambda_{i}$, the max-min represents maximizing the minimum coupling efficiency at wavelength $\lambda_{\min}$ within the target band. The multi-objective algorithm can optimize the metasurface structure at the wavelength with the lowest coupling efficiency, enhancing the similarity between the metasurface-excited near fields and the waveguide eigenmodes. The selection of coupling mode is an adaptive result of the algorithm, while the design of objective functions also enables effective selection of the target coupling modes. ^[37]^

The minimum mesh step was set as 2.5 nm considering the trade-off between simulation time and accuracy. The optimization simulation was carried out on a workstation with Intel Gold 6256 CPU and each simulation used 24 cores. For single-wavelength optimization and broadband optimization, the number of iterations is set to 100 and 500, respectively, in order to search for the local optimal solutions with better performance. Particularly, each iteration of single wavelength topology optimization takes ≈ 200 s, including both forward and adjoint simulations. For broadband optimization conditions, each iteration takes ≈ 250 s, including both forward and adjoint simulations. Time-domain forward simulation, as shown in Figure S7 (c), can perform parallel computation of forward field distributions at 100 wavelength points across the 1250 nm to 1650 nm bandwidth, with the increase in computational time being much smaller compared to conventional multi-objective algorithms. In the subsequent numerical computations, it is straightforward to identify the wavelength positions with the lowest efficiency and perform adjoint simulation at those specific points. Therefore, the time and computational resources required for the adjoint process is comparable to that of a single-objective optimization process. Overall, time-domain forward simulation provides an approach for broadband multi-objective optimization that can significantly save both computational time and resources.

**Supplementary Note 8: Near-field distribution at different wavelength for forward and inverse designed quasi-continuous meta-couplers.**

For the optimized quasi-catenary structure shown in Figure 4 (a), the near-field distributions at different wavelengths are illustrated in Figure S8 (b) based on the schematic introduced in Note 3. As can be seen, the adjacent coupling and inside field distribution show great differences for initial and optimized conditions at each wavelength. The destructive interference for conventional catenary conditions has been optimized. The intensity of the electromagnetic field beneath the metasurface region is efficiently enhanced around the target 1550 nm wavelength. Furthermore, the spurious diffraction order is effectively suppressed at 1550 nm leading to a high ER. The *n*_eff_ curve and far-field distributions of the optimized structure are derived using a similar method mentioned in Note 4 and Note 5.


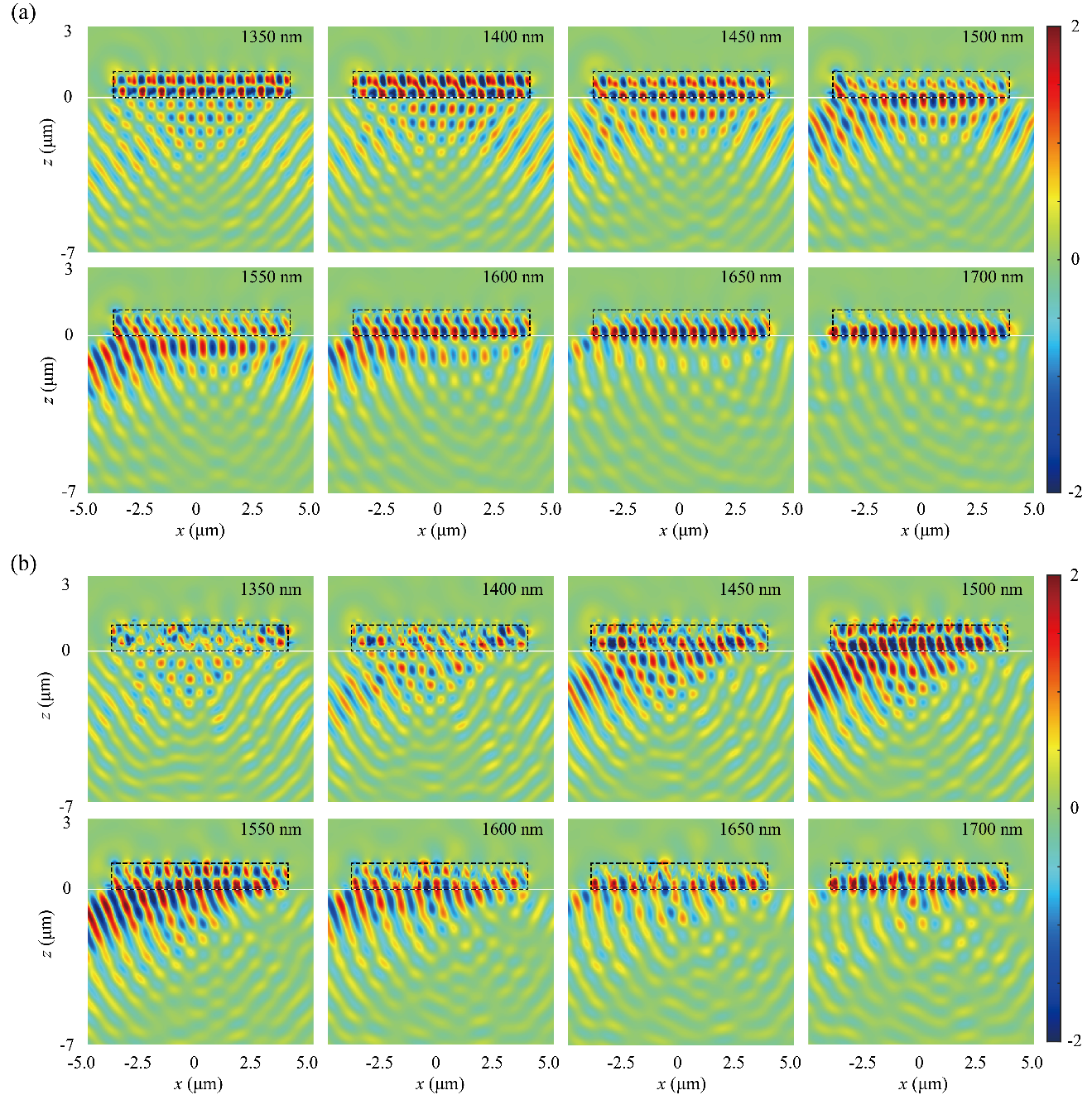


**Figure S8.** (a) Near-field E*_z_* distributions at different wavelengths for meta-coupler with nine catenary structures; (b) Near-field E*_z_* distributions at different wavelength for meta-coupler with optimized structures. The optimization wavelength is 1550 nm.

**Supplementary Note 9: Near-field distribution at different wavelength within the O-, S-, C- and L-band for forward and inverse designed quasi-continuous meta-couplers.**

Using the same near-field analysis strategy mentioned above, the near-field Ez distributions at different wavelength for meta-coupler with broadband optimized structures can be obtained. As the incident wavelength varies, the local electric field distribution within the metasurface gradually changes, which is attributed to the mode dispersion behavior of the free-form metasurface. Moreover, the inverse-design algorithm can identify the eigenmodes most similar to the near-field distributions at different wavelengths and modify corresponding near-field distributions. Therefore, the mode-matching conditions can be satisfied across a wide spectral range leading to broadband coupling. The inverse-design algorithm adaptively controls the near-field mode dispersion behavior within the metasurface. As can be seen, the unidirectional diffractions with near uniform efficiency at the entire band covering the O- to L-bands are effectively implemented. Moreover, the spurious diffraction order is effectively suppressed.


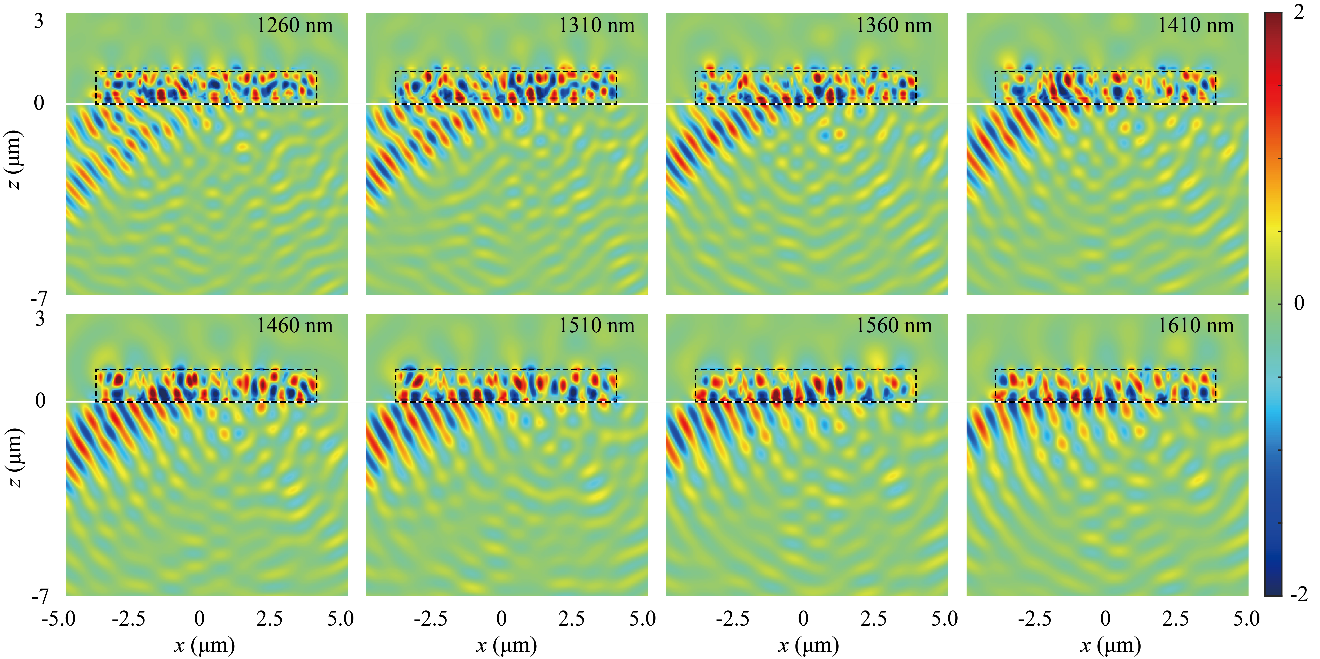


**Figure S9.** Near-field E*_z_* distributions at different wavelength for meta-coupler with broadband optimized structures. The optimization band is 1260 to 1625 nm.

It is worth noting that the metasurface integrated on top of the waveguide coupler in this work is constructed with amorphous silicon. To achieve sufficient phase modulation capability, the refractive index of the metasurface must be adequate. Otherwise, the modulation depth would need to be extended, which is not conducive to integration and fabrication. Therefore, the high index contrast between the silicon metasurface and the Si_3_N_4_ waveguide is favorable for efficient antenna excitation. Additionally, the low loss at the communication wavelength of 1550 nm and the mature fabrication processes of these two materials are also reasons for their selection. ^[35, 36]^ As mentioned in the main text, the full model topology optimization method can be extended to different waveguide platforms, such as the coupling between two waveguides or between a waveguide and an optical fiber. ^[20]^

**Supplementary Note 10: Numerical Simulations.**

The finite-difference time-domain (FDTD) technique is utilized to conduct full-wave numerical simulations, encompassing amplitude distributions and mode profiles. The refractive index of silicon, sourced from Palik data for amorphous silicon, is set at 3.47 for a wavelength of 1550 nm. A total-field-scattered-field (TFSF) source is used to simulate the normally incident plane wave on the metasurface. The field in a volume encompassing the patterned metasurface, waveguide, and the substrate was simulated and terminated on all sides by perfect matched layer boundary condition. An automatic meshing procedure is implemented to ensure accurate meshing at the interface. The minimum mesh step is set to 2.5 nm, balancing simulation time and accuracy. The optimization simulation was carried out on a cluster with Intel Gold 6256 CPU and each simulation used 24 cores. Particularly, each of iteration of topology optimization took around 200 s, including both forward and adjoint simulations. A time-domain simulation procedure is employed for the forward simulation of broadband optimization to save time. Therefore, broadband optimization iteration is also around 200 s. Max-min gradient is utilized to optimize lowest coupling efficiency at a single wavelength.

**Supplementary Note 11: Proposed fabrication procedure.**

The meta-waveguide coupler consists of a Si_3_N_4_ waveguide structure, an amorphous silicon upper layer, and a SiO_2_ substrate. The designed structure can be obtained using a two-step electron beam lithography (EBL) and etching procedure. Initially, a 1000 nm Si_3_N_4_ film is deposited on the SiO_2_ substrate using plasma-enhanced chemical vapor deposition (PECVD). This film is then selectively etched to create the waveguide structure. To protect the waveguide, a 30 nm layer of Al_2_O_3_ is deposited on the Si_3_N_4_ waveguide via electron beam evaporation. Subsequently, a 1000 nm-thick amorphous silicon layer is deposited using PECVD. Photoresist is then spin-coated, and EBL is applied to define the desired photoresist pattern. Inductively coupled plasma reactive ion etching, utilizing an SF_6_ and C_4_F_8_ gas mixture, is employed to etch the silicon structures. Finally, the top photoresist layer is removed to yield the freeform metasurface.

**Supplementary Note 12: Analysis of feature size robustness.**

Furthermore, we have established a numerical simulation model for the fabrication error analysis. In this section, we introduce the dilated and eroded model to modify three free-form meta-atoms in the optimized structure for the high-efficiency and broadband coupling conditions. Figure S10 (a) and Figure S11 (a) illustrates three structures that contain sub-100 nm features as examples, as shown in Areas 1, 2, and 3. Simulated fabrication errors from -30 nm to +30 nm are generated using MATLAB and introduced into all sub-100 nm areas including these three regions. As can be seen from the comparisons of coupling efficiency spectra and mode purity spectra, the efficiency reduction increases with the eroded error getting larger, as shown in Figure S10 (b) and Figure S11 (b). However, for dilated conditions, the meta-coupler devices exhibit great broadband or high-efficiency performance with the dilated error increases, as shown in Figure S10 (c) and Figure S11 (c). Furthermore, the mode purity exhibits a negligible sensitivity to variations in feature size. These results indicates that during the fabrication process, the small feature regions can be optimized through dilated model, thereby accommodating the requirements of actual fabrication precision. Therefore, it is verified that optimized free-form meta-coupler can tolerate approximately -10 nm to +30 nm of fabrication errors while maintaining excellent device performance, demonstrating robust feasibility for practical device fabrication.


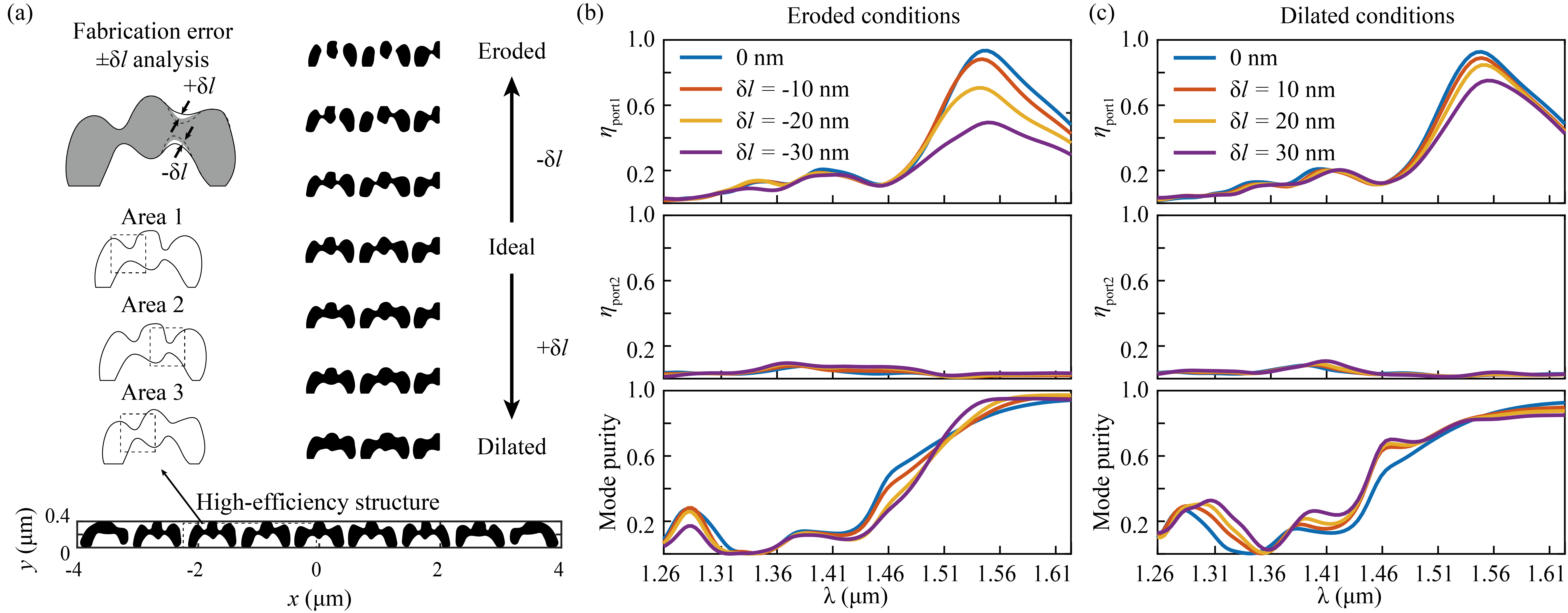


Figure S10. (a) Initial metasurface structure for the high-efficiency coupling condition and three structures with small feature size existed in Area 1,2, and 3; (b) Coupling efficiency spectrum at two ports and mode purity spectrum at port1(left port) for the eroded model; (c) Coupling efficiency spectrum at two ports and mode purity spectrum at port1(left port) for the dilated model;


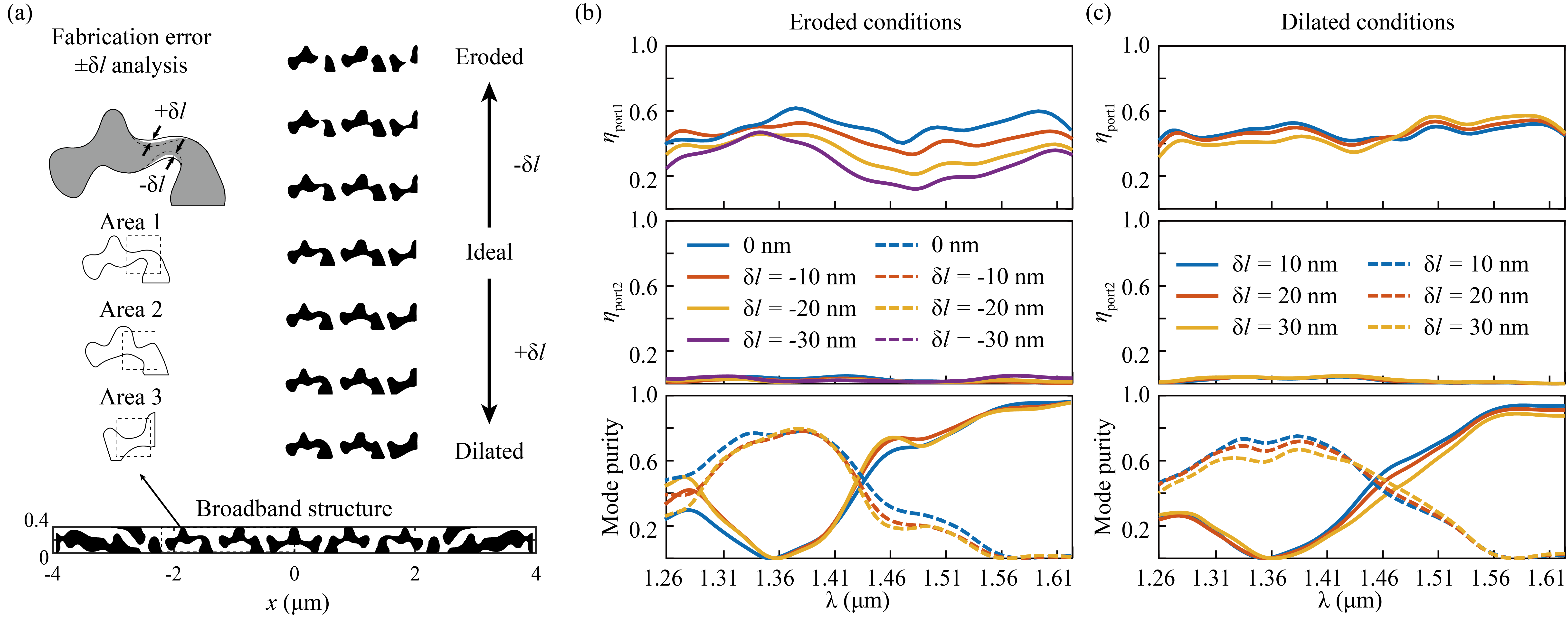


Figure S11. (a) Initial metasurface structure for the broadband coupling condition and three structures with small feature size existed in Area 1,2, and 3; (b) Coupling efficiency spectrum at two ports and mode purity spectrum at port1(left port) for the eroded model; (c) Coupling efficiency spectrum at two ports and mode purity spectrum at port1(left port) for the dilated model. Dashed lines represent mode purity spectrum for TM01 modes and solid lines represent mode purity spectrum for TM00 modes.

**Supplementary Note 13: Analysis of the impact of incident beam position and size.**


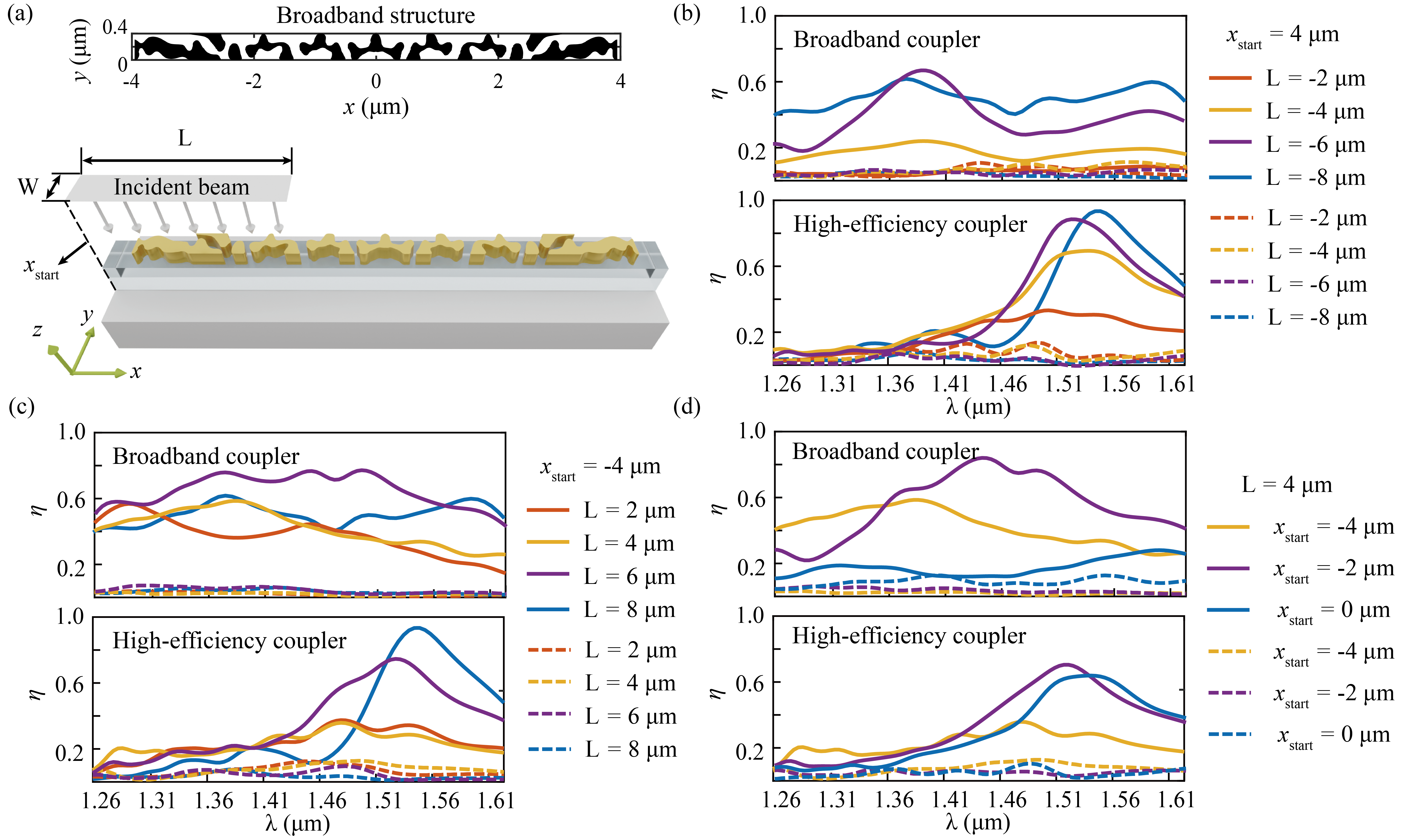


Figure S12. (a) Meta-coupler illuminated with incident beam of varying size and location. In where the L, W, and *x*_start_ represent the length, width, and start position on the *x*-axis, respectively; (b) Coupling efficiency spectrum as a function of L at two ports for high-efficiency and broadband couplers. In which, the W and *x*_start_ are 0.4 $\text{μm}$and 4 $\text{μm}$, respectively; (c) Coupling efficiency spectrum as a function of L at two ports for high-efficiency and broadband couplers. In which, the W and *x*_start_ are 0.4 $\text{μm}$and -4 $\text{μm}$, respectively; (d) Coupling efficiency spectrum as a function of *x*_start_ at two ports for high-efficiency and broadband couplers. In which, the W and L are 0.4 $\text{μm}$and 4 $\text{μm}$, respectively; The dashed line represents coupling efficiency at the opposite port (right port).

The free-form meta-coupler devices obtained in this work are designed under full-model simulation conditions and multiple reflections of the guided mode within the waveguide can result in repeated interactions with the metasurface. Therefore, it is essential to evaluate the impact of incident beam displacement on the device performance. The repeated interactions of the optical field can be controlled by adjusting the beam incident position. When the beam is only incident on the right end (*x*_start_ = 4 $\text{μm}$), the left-end structure interacts only with the internal reflected field. When the beam is only incident on the left end (*x*_start_ = -4 $\text{μm}$), the left-end structure interacts only with the incident beam. Figure S12(a) illustrates the schematic of the coupler device illuminated by incident optical fields of varying size and position. A total-field-scattered-field (TFSF) source is used to simulate the normally incident plane wave on the metasurface. ^[36]^ As shown in Figure S12(b-d), the impact of position and beam size on the broadband coupler primarily affect the coupling efficiency, while the broad bandwidth features are preserved in most cases. In contrast, the high-efficiency coupler experiences a significant reduction in peak efficiency when the source size is smaller than 4 $\text{μm}$ or when the source is positioned far from the center position of the metasurface. The coupling peak at 1550 nm can only be preserved under conditions with small size errors ($\left|L\right|\geq4\text{μm}$). It is worth noting that the device performs better when the light source is positioned away from the left port, compared to when the source is at the left (*x*_start_ = -4 $\text{μm}$), as shown in Figure S12 (b) and (d). This is because the quasi-continuous structures at both ends of the metasurface are generated when the guided mode and the incident electric field coexist. This phenomenon also demonstrates that full-model inverse design algorithm can effectively account for the multiple reflections within the waveguide.
